# Auxin‐related regulation of barley floral organ fate: genetic and phenotypic insights from *tweaky spike* mutants

**DOI:** 10.1111/nph.71533

**Published:** 2026-09-09

**Authors:** Vėjūnė Pukenytė, Bernadeta Kaminskaitė‐Mačė, Miglė Remeikytė, Jurga Jankauskienė, Julius Juodakis, Sigita Jurkonienė, Algė Leistrumaitė, Ljudmilla Timofejeva, Virginija Vaitkūnienė, Justas Stonkus, Raimondas Šiukšta

**Affiliations:** ^1^ Institute of Bioscience, Life Sciences Center Vilnius University LT‐10257 Vilnius Lithuania; ^2^ Laboratory of Plant Physiology, Nature Research Centre Akademijos Str. 2 08412 Vilnius Lithuania; ^3^ Institute of Agriculture, Lithuanian Research Centre for Agriculture and Forestry Institutas Ave. 1, Akademija 58344 Kėdainiai Lithuania; ^4^ Centre of Estonian Rural Research and Knowledge Jõgeva Township 48309 Estonia; ^5^ Institute of Biochemistry, Life Sciences Center, Vilnius University LT‐10257 Vilnius Lithuania

**Keywords:** AP2L transcription factor, auxin homeostasis, barley (*Hordeum vulgare*), barley ABC flower development model, floret development, HvAP2L5, inflorescence development

## Abstract

- Mutant‐based research is a cornerstone for elucidating gene function in barley (*Hordeum vulgare*) and other cereals. In this study, we exploited a unique collection of barley *tweaky spike* (*tw*) mutants to identify the locus controlling floret and spike development.
- We combined genetic mapping with structural variant and gene‐expression analyses across a *tw* allelic series, supported by functional validation, and quantified endogenous auxin levels alongside auxin‐application assays to test phytohormone involvement.
- Analysis of independent *tw* allelic mutants revealed that the phenotype results from loss of function of *HvAP2L5*, most often due to complete open reading frame (ORF) deletions, and, in one allele, due to transcriptional silencing. *tw* mutants showed a reproducible endogenous indole‐3‐acetic acid deficit, and exogenous auxin could partially phenocopy and/or rescue core *tw‐*associated traits, indicating compromised auxin homeostasis.
- We conclude that *HvAP2L5* is essential for normal spike/floret development and that its loss disrupts auxin balance. Auxin imbalance is a compelling working model that can account for most of the pleiotropic features of *tw* mutants.

## Graphical abstract

Inflorescence/floret phenotypes of *tweaky spike (tw) barley* (*Hordeum vulgare*) mutants and their respective WT backgrounds.

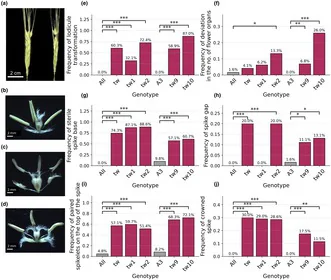

## Introduction

Inflorescence architecture strongly influences cereal yield because it determines the number of spikelets and florets, and thus grain number per spike or panicle (Yuan *et al*., 2020; Zhang *et al*., 2024). In barley (*Hordeum vulgare*), reproductive development begins when the shoot apical meristem (SAM) transitions into an inflorescence meristem (IM). The IM then produces spikelet primordia along the rachis: at each node, a triple spikelet meristem is initiated and differentiates into one central and two lateral spikelet meristems (Koppolu & Schnurbusch, 2019; Thiel *et al*., 2021). Beyond spikelet initiation, barley spike development requires coordinated control of spikelet meristem fate (e.g. determinacy vs branching), rachilla growth and termination, and floral organ specification within each floret (Sakuma & Koppolu, 2023).

Recent studies in barley and other Triticeae have emphasized that meristem activity and determinacy are not controlled by a single genetic switch, but rather by multiple, partially independent regulatory modules that govern (1) how long the IM remains active and how many spikelet primordia it initiates (e.g. CENTRORADIALIS [CEN], CLAVATA1 [CLV]‐FON2‐LIKE CLE PROTEIN1 [FCP1] signaling, FASCIATED EAR [FEA], APETALA2‐like transcription factor (AP2L‐H5) (Bi *et al*., 2019; Zhong *et al*., 2021; Wang *et al*., 2023; Zhang *et al*., 2024; Vardanega *et al*., 2025), (2) when and where spikelet primordia are specified from the IM (e.g. Six‐rowed spike2 [VRS2]/Six‐rowed spike4 [VRS4]/COMPOSITUM1 [COM1]/COMPOSITUM2 [COM2]) (Koppolu *et al*., 2013; Poursarebani *et al*., 2015, 2020; Youssef *et al*., 2017), and (3) how the spikelet meristem transitions to floret development and arrests rachilla growth (e.g. APETALA 2‐like genes, *Multiflorus*) (Debernardi *et al*., 2020; Koppolu *et al*., 2022). Such findings reinforce a broader principle in grasses: relatively small shifts in the timing or strength of these meristem‐maintenance and identity pathways can generate large architectural outcomes, including changes in spikelet density, spike and spikelet determinacy, and floral complexity (Bommert & Whipple, 2018).

At the level of the floret, barley develops a highly specialized set of organs. Each floret is enclosed by the lemma and palea and typically contains two lodicules, three stamens, and a central pistil (Sakuma & Koppolu, 2023). Lodicules are small, grass‐specific organs homologous to petals; their rapid swelling at anthesis forces the lemma and palea apart, enabling floret opening and pollen release (Nair *et al*., 2010; Anwar *et al*., 2018). The interaction of canonical A, B, and C class transcription factors specifies the identity of these floral organs. In barley florets, the first whorl comprises the outer, bract‐like organs lemma and palea, whose identity is largely specified by A‐class genes activity mediated by *AP1/AP2*‐type regulators. The second whorl is formed by the grass‐specific lodicules and requires the combined functions of A‐ and B‐class genes for proper specification. B and C jointly specify stamen fate, and C confers carpel identity in the fourth whorl (Krogan *et al*., 2012; Callens *et al*., 2018; Kuijer *et al*., 2021). Consistent with an important role of A‐class regulators in lodicule development, *Cleistogamy 1* (*Cly1*), encoding an AP2‐like transcription factor (ortholog of *Arabidopsis thaliana AP2*), restricts lodicule growth in barley: a miR172‐sensitive allele allows normal swelling, whereas the miR172‐resistant allele (*cly1.b*) results in atrophic, nonswelling lodicules and cleistogamy (Nair *et al*., 2010). Similarly, the AP2 gain‐of‐function allele *Zeo1.b* produces small lodicules that fail to swell at anthesis (Houston *et al*., 2013).

Auxin is a central morphogen signal in plant development, establishing local concentration maxima at sites where floral and inflorescence organ primordia are initiated. These auxin peaks promote meristem specification and help coordinate organ initiation with organ‐identity programs (Zhao, 2018; Yuan *et al*., 2020; Lu *et al*., 2024; Vanneste *et al*., 2025). Importantly, altered auxin levels can also influence floral organ identity: in rose, elevated auxin promotes a petal‐to‐stamen shift via Auxin Response Factor (ARF)‐mediated regulation of C‐class gene expression, highlighting the capacity of auxin to remodel petal‐homologous tissues (Chen *et al*., 2021). Although there is evidence that auxin homeostasis contributes to proper petal, stamen, and carpel formation in the Rosaceae (Chen *et al*., 2021; Lu *et al*., 2024), how this relationship is established and deployed during floret development in cereals remains largely unexplored.

Mutant collections remain a cornerstone of cereal developmental genetics. Both classical and modern mutagenesis generate allelic series that help link visible architectural changes (such as altered meristem determinacy, spikelet initiation, rachilla activity, or floral organ identity) to specific developmental pathways (Lundqvist & Franckowiak, 2003; Terzi *et al*., 2017; Hansson *et al*., 2024). A series of unique recessive pleiotropic barley mutants, *tweaky spike* (*tw*), with disturbed inflorescence development, were created in the Department of Botany and Genetics of Vilnius University. All *tw* mutants are characterized by a shortened spike with sterile spikelets at the basal part and hyperdeveloped spikelets at the apical region (forming a crown), unstable transformation of lodicules into reproductive organs, increased susceptibility to fungal infections, and altered quantitative and qualitative grain characteristics. Phenotype‐rescue experiments demonstrated that the *tw* mutation disrupts auxin physiology, which is likely responsible for the phenotypic instability observed in hybrids between *tw* and other homeotic mutants (Žvingila *et al*., 2012; Šiukšta *et al*., 2015, 2018, 2021). Despite their pleiotropic effects and reduced fertility, *tw* mutants serve as valuable material for dissecting the genetic and hormonal regulation of inflorescence development. In this study, we leverage the unique allelic series of *tw* mutants to (1) explore *tw* locus influence in inflorescence determinacy and lodicule fate, and (2) test how altered auxin homeostasis contributes to the expressivity and instability of the tw phenotype.

## Materials and Methods

### Plant material and phenotyping

Recessive pleiotropic *tw* mutants of barley (*Hordeum vulgare* L.) were obtained in three independent chemical mutagenesis experiments. The first *tw* mutant was induced in 1977 by treating grains of the cultivar (cv) ‘Auksiniai II’ (AII) with ethylenimine and detergent. Subsequently, two additional independent mutants, *tw1* and *tw2*, were induced in the same cultivar (‘Auksiniai II’) following phenobarbital treatment. In a third mutagenesis experiment, nine independent mutants (*tw3*–*tw11*) were generated in the cv ‘Auksiniai 3’ (A3) after combined treatment with ethylenimine, cysteine, and para‐aminobenzoic acid. The *tw* mutant collection was originally generated by Prof. Vytautas Rančelis at Vilnius University.

The *tw* mutant stocks and their respective wild‐type (WT) controls (cv ‘Auksiniai II’ (AII), and cv ‘Auksiniai 3’ (A3)) were obtained from the Vilnius University Botanical Garden collection.

The full collection contains *tw*, *tw1*, *tw2* (possessing the AII background) and *tw4*, *tw5*, *tw6*, *tw8*, *tw9*, *tw10* (possessing the A3 background). While the full collection was used for candidate gene validation, only a subset (*tw*, *tw1*, *tw2*, *tw9*, *tw10*, and their corresponding WTs AII and A3) was used for morphometric trait measurements.

Morphometric traits – plant height, tiller number per plant, spike length, awn length, sterile spikelets per spike, and grain number per spike – were assessed across three consecutive seasons (2021–2023). Field experiments were conducted on Endocalcari–Epihypogleyic Cambisol with near‐neutral pH, moderate levels of plant‐available phosphorus and potassium, and a humus content of 2.25 g kg^−1^. Each plot measured 1.5 × 10 m and was sown at a density of 450 grains per m^2^. The trials followed a randomized complete block design with two replicates. Standard crop management practices were applied in all seasons, including presowing N–P–K fertilization at 90–60–60 kg ha^−1^ and herbicide and insecticide treatments when required. For quantitative trait assessment, 30 plants or spikes were analyzed per line (Vaitkūnienė *et al*. 2019).

### Barley hybridization

To generate F_2_ mapping populations, two crosses were performed under the field conditions: *tw* × AII and *tw2* × AII. The F1 generation was grown in greenhouse (16 h : 8 h, light : dark). F_2_ populations were cultivated under field conditions at the Vilnius University Botanical Garden. Plants were classified based on the presence or absence of the tw phenotype, defined as at least one tiller bearing a crowned spike (classified as *tw*), while those lacking any crowned spikes were scored as WT.

### Genomic DNA extraction and whole‐genome sequencing

Genomic DNA was extracted using a modified hexadecyltrimethylammonium bromide (CTAB) protocol (Doyle & Doyle, 1990) from 46 AII WT plants, 50 tw phenotype F_2_ plants from the *tw* × AII cross (*tw‐F*_*2*_), and 50 tw phenotype F_2_ plants from the *tw2* × AII cross (*tw2‐F*_*2*_). Four DNA samples were prepared for whole‐genome sequencing (WGS): (1) AII individual – DNA from a single AII plant, (2) AII pool – DNA equally mixed from 46 AII individuals, (3) *tw* pool – DNA equally mixed from 50 *tw‐F*_*2*_ plants, and (4) *tw2* pool – DNA equally mixed from 50 *tw2‐F*_*2*_ plants. DNA samples were submitted to Novogene (UK) for WGS on the Illumina Novaseq X Plus PE150 platform. Each library yielded *c*. 80–92 Gb of sequence data, corresponding to > 20× coverage of the barley genome.

### WGS data processing, mapping, and SNP visualization

The quality of the raw sequencing reads was assessed using FastQC. Adapter trimming and quality filtering were performed using Cutadapt 3.5 (Martin, 2011). Cleaned reads were re‐assessed with FastQC to ensure quality standards were met. Reads were mapped to the Morex V3 barley reference genome (Mascher *et al*., 2021) using BWA 0.7.17‐r1188 (Li & Durbin, 2009). Single‐nucleotide polymorphisms (SNPs) were called jointly from the AII individual and the two mutant F_2_ pools (*tw‐F*_*2*_ and *tw2‐F*_*2*_) using bcftools 1.13 (Danecek *et al*., 2021). SNP‐index values were calculated, and allele frequency plots were generated using the MutMap pipeline v.2.3.4 (Abe *et al*., 2012), with a WT individual serving as a reference to define SNP calls and eliminate background polymorphisms. Nonrecombining regions were identified as genomic intervals in which the SNP‐index of the mutant bulk consistently approached 1.0.

### SNP filtering

After identification of the nonrecombining genomic regions, variant calling for each sample (in the 1H: 3.2–6.8 Mb, 5H: 530–580 Mb regions) was performed individually using bcftools 1.13. The resulting VCFs were annotated with predicted variant effects using SnpEff (Cingolani *et al*., 2012). A custom filtering pipeline was applied to extract relevant variant information. This output was then imported into R 4.5.2 for further analyses. For the *tw‐F*_*2*_ and *tw2‐F*_*2*_ mutant pool, SNPs were filtered to retain only high‐confidence variants, requiring a minimum allele frequency of 0.98 (allowing 2% sequencing error), read depth (DP) of at least five reads (DP > 5), mapping quality > 30 (MQ > 30); only variants annotated as moderate or high impact (based on SnpEff) were retained for further analysis.

For the AII WT pool, SNPs were filtered only for coverage and quality (DP > 5, MQ > 30), retaining all variants regardless of frequency to facilitate background subtraction. Shared variants between mutant pools and the AII datasets were removed.

The remaining candidate SNPs were linked to specific genes using SnpEff annotation. Genes with high‐impact SNPs detected in both *tw* and *tw*_*2*_ pools were prioritized. To further assess the functional relevance of retained missense mutations, amino acid substitutions were manually submitted to the Sorting Intolerant From Tolerant (SIFT) prediction tool (Ng & Henikoff, 2003) to evaluate potential protein impact. SNPs predicted as ‘tolerated’ were excluded from the candidate gene list.

### Structural genome variant analysis

To identify structural variants potentially associated with the tw phenotype, read alignments from mutant and WT samples were processed and analyzed using dysgu (Cleal & Baird, 2022). Binary Alignment Map (BAM) files were first prepared by correcting mate‐pair information, sorting by genomic position, marking PCR duplicates, and indexing. Structural variant calling was performed individually for each sample using the dysgu run module, with the Morex V3 genome as the reference; then, variants were filtered using AII individual calls as normal. The resulting files were examined for deletions and other large‐scale rearrangements affecting gene‐coding regions within the candidate interval defined by MutMap. Candidate genes were evaluated in all allelic *tw* mutants using PCR and Sanger sequencing (Azenta Life Sciences, Germany). The primers used are listed in Supporting Information Table S1.

### Total RNA extraction and cDNA synthesis

Fifty seeds from the AII, *tw*, and *tw1* genotypes were germinated on filter paper for 3 d at room temperature in the dark. Twelve seedlings were transferred to soil pots and grown in a growth chamber under long‐day conditions (16 h : 8 h, light : dark, 20°C).

Samples were collected in three biological replicates: coleoptiles (up to 4 d after germination), roots of 4‐d‐old seedlings, leaves from plants at the 5^th^ leaf stage, when inflorescence development initiates in seedlings (Yagil & Stebbins, 1968), and young spikes from 37‐d‐old plants (*c*. 1 cm long; one sample consisted of 4 spikes). All collected samples were immediately frozen in liquid nitrogen, homogenized, and used for total RNA extraction.

Total RNA was extracted from all collected samples using the innuPREP Plant RNA kit (IST Innuscreen GmbH, Germany) following the manufacturer's protocol. The residual DNA from the total RNA samples was eliminated using the innuPREP DNase I Digest Kit (IST Innuscreen GmbH). The quality and concentration of the extracted RNA were evaluated using 1.5% agarose gel electrophoresis and NanoDrop spectrophotometer (NanoDrop™ 2000, Thermo Fisher Scientific, Wilmington, DE, USA). Immediately after the RNA extraction, cDNA was synthesized using the High‐Capacity cDNA Reverse Transcription Kit with an RNase Inhibitor (Thermo Scientific, Vilnius, Lithuania) according to the manufacturer's protocol. cDNA samples were diluted 1 : 20, aliquoted, and stored at −80°C for gene expression assays, then used without refreezing.

### Quantitative PCR assays for Gene Expression Analysis

Gene expression changes in different developmental stages were determined using the Maxima SYBR Green/ROX qPCR Master Mix Kit (Thermo Scientific). The ADP‐ribosylation factor 1–like protein housekeeping gene (*HvADP*) and noncoding small nucleolar RNA 14 (snoR14) were used as endogenous controls (Ferdous *et al*., 2015). The sequences of the quantitative polymerase chain reaction (qPCR) primers used are listed in Table S2. All qPCRs were performed on a SYBR Green fluorescence system using a QuantStudio 3 Real‐Time PCR System (Applied Biosystems, Singapore). The geometric mean of the expression levels of two reference genes was used to normalize target gene expression (Vandesompele *et al*., 2002). Relative expression of the *HvAP2L5* gene across developmental stages and of auxin‐related genes (differentially expressed in *HvAP2L5* mutants according to Zhong *et al*. (2021)) in the WT and the *tw* and *tw1* mutant genotypes was calculated as 2^−ΔCt^, where $\Delta \mathrm{Ct}={\mathrm{Ct}}_{\text{target}}-{\mathrm{Ct}}_{\text{reference}}$.

### Phytohormone treatment

The influence of phytohormones on floral and spike development in barley was investigated by treating both WT and *tw* mutant plants with synthetic analogues of three phytohormone classes: gibberellins (GA), cytokinins (CK), and auxins (AUX). The treatments were performed by spraying phytohormones at the early floral initiation stage (5th leaf stage) using GA3 (100 mg l^−1^ GA3, 0.1% Tween 20), 6‐benzylaminopurine (BAP) (100 mg l^−1^ BAP, 0.1% Tween 20), and 2,4‐dichlorophenoxyacetic acid (2,4‐D) (2 g l^−1^ 2,4‐D, 0.1% Tween 20) as respective phytohormone analogues. Since the *tw* mutants are characterized by four distinctive phenotypic traits: spike tips forming crown‐like structures, spikeletless rachis node/elongated internode (spike gap), lodicule to reproductive organ transformations, and deviation in the number of floral organs, the effect of phytohormone treatments was assessed by quantifying the frequency of these phenotypic features in fully developed spikes.

### Extraction of free IAA and its conjugates

To measure the levels of indole‐3‐acetic acid (IAA) and its conjugated forms (IAA‐Asp, IAA‐Glu, IAA‐Leu, IAA‐Val, IAA‐Phe, IAA‐glucose) in barley tissue, plant material was extracted with 80% cold methanol containing an antioxidant (BHT, 10 mg l^−1^). The extraction was carried out in the dark at +4°C for 14 h, using a 1 : 10 ratio of fresh plant tissue to extraction solvent. After extraction, the methanolic solution was filtered through a 0.2 μm nitrocellulose membrane (Whatman, Germany). Phenolic compounds were removed from the filtrate using polyvinylpyrrolidone (PVP, Serva, Germany), and the resulting extract was concentrated to an aqueous phase using a vacuum evaporator (IKA RV 10 Basic, Germany).

For the separation of free IAA and its conjugates, standard liquid–liquid extraction was performed using ethyl acetate at a pH below 3.0, following the method described by Normanly (2010) for indole compound fractionation.

### Identification and quantification using HPTLC

IAA and its conjugates were identified and quantified in coleoptiles by high‐performance thin‐layer chromatography (HPTLC) on a CAMAG system (Switzerland) using the visionCATS 2.5 software. Samples and standards were applied to 20 × 10 cm silica gel TLC plates in 6‐mm‐wide bands using a TLC 4 automated sampler and a 15 μl dosing syringe (Hamilton, USA). Chromatographic separation was performed in an Automatic Developing Chamber 2 using a mobile phase of isopropanol, ammonia, and water (8 : 1 : 1).

After development, the plates were dried and visualized under UV light at 254 nm and 366 nm, as well as under white light, using the TLC Visualizer 2. Absorbance measurements were taken using the TLC Scanner 4. Peak areas of sample bands were compared to those of known standards, and the concentrations of free IAA and its conjugates were calculated using calibration curves based on reference indole compounds.

### Identification and quantification using LC‐MS/MS

Plant extracts from developing spikes (spikes were collected from 37‐d‐old plants, for analysis selected only spikes that were less than 1 cm long, one sample consisted of 4 spikes) were analyzed by liquid chromatography–tandem mass spectrometry using a Nexera X2 UHPLC system coupled to an LC‐MS‐8050 mass spectrometer (Shimadzu, Japan) equipped with an ESI source. Chromatographic separation was carried out using a 3 × 150 mm YMC‐Triart C18 (particle size 3 μm) column (YMC, Japan, #TA12S03‐1503WT) at 40°C. The mobile phase consisted of 0.1% formic acid (solvent A) and acetonitrile (solvent B) delivered in gradient elution mode at a 0.45 ml min^−1^ flow rate. The following elution program was used: 0 to 1 min, 5% solvent B; 1 to 5 min, 95% solvent B; 5 to 7 min, 95% solvent B; 7 to 8 min, 5% solvent B; 8 to 12 min, 5% solvent B. IAA and IAA conjugates were detected using ion transitions as follows: 176 > 130(+) for IAA, 291 > 130(+) for IAA‐Asp, 305 > 130(+) for IAA‐Glu, 289 > 130(+) for IAA‐Leu, 275 > 130(+) for IAA‐Val, 323 > 130(+) for IAA‐Phe, and 336 > 130(−) for IAA‐glucose at an interface temperature of 300°C and desolvation line temperature of 250°C. Nitrogen was used for nebulizing (3 l min^−1^) and drying (10 l min^−1^). Dry air was used for heating (10 l min^−1^). The collected data were analyzed using the LabSolutions software v.5.82 SP1 (Shimadzu, Japan). The amounts of free IAA and IAA‐conjugates were determined from the peak areas by using external calibration curves for quantification and sample‐to‐sample comparison.

### Plasmid construction and plant transformation

*tw.c1* mutant allele was generated using CRISPR‐Cas9 genome editing. All sgRNAs were designed using the CRISPOR (Concordet & Haeussler, 2018) software (Table S1). Hybridized oligos for target 1 or 3 (T1 and T3) were ligated into plasmid pHvL1P3GA (AddGene #112027), while oligos for T2 or T4 were ligated into pHvL1P4GA (AddGene #112028) according to the protocol of Lawrenson & Harwood (2019). Then, the GoldenGate cassettes expressing plant hygromycin resistance (pICSL11059, AddGene #68263), Cas9 (pICSL11056, AddGene #68258) (Lawrenson *et al*., 2015), and two gRNA cassettes (pHvL1P3GA, pHvL1P4GA (Lawrenson & Harwood, 2019)) were cloned into binary vector pAGM8031 (AddGene #48037, Weber *et al*. (2011)). Immature embryos from ‘Golden promise’ were transformed using *Agrobacterium tumefaciens* strain AGL1 according to Hinchliffe & Harwood (2019). For callus induction (CI), embryos were grown on CI medium (Hinchliffe & Harwood, 2019) at 24°C in the dark for 6 wk, with medium changed every 2 wk. Proliferated calluses were then transferred to barley regeneration medium (BMR) medium (Marthe *et al*., 2015) and grown at 24°C under 80% humidity, a 16 h : 8 h, light : dark photoperiod, with BMR medium replaced fortnightly. After shoots reached 2–3 cm, they were transferred to tubes containing BMR medium for rooting. Once the roots developed, they were potted in soil and grown in the greenhouse. T‐DNA insertion was validated by DNA extraction and PCR (primer targeting *hygromycin phosphotransferase II* (*hpt*) gene (Table S1)) and *HvAP2L5* gene changes were validated using Sanger sequencing (Azenta Life Sciences, Germany).

### Statistical analysis

Comparisons between each *tw* allele and its corresponding WT for continuous traits were performed using the Mann–Whitney U test, followed by Benjamini–Hochberg correction for multiple comparisons. Differences in phenotype frequencies were assessed using a two‐proportion Z‐test.

## Results

### The *tw* mutation affects morphometric traits in barley

Morphometric traits of *tw* barley mutants were evaluated over three consecutive years (2021, 2022, 2023). Measured traits, including plant height, tiller count, spike length, awn length, number of sterile spikelets per spike, and grain count per spike, are shown in Figs 1, S1; Tables S3, S4; Dataset S1. Over the three years, *tw* lines exhibited reproducible and highly significant differences from WT (Fig. S1).

**Fig. 1:**
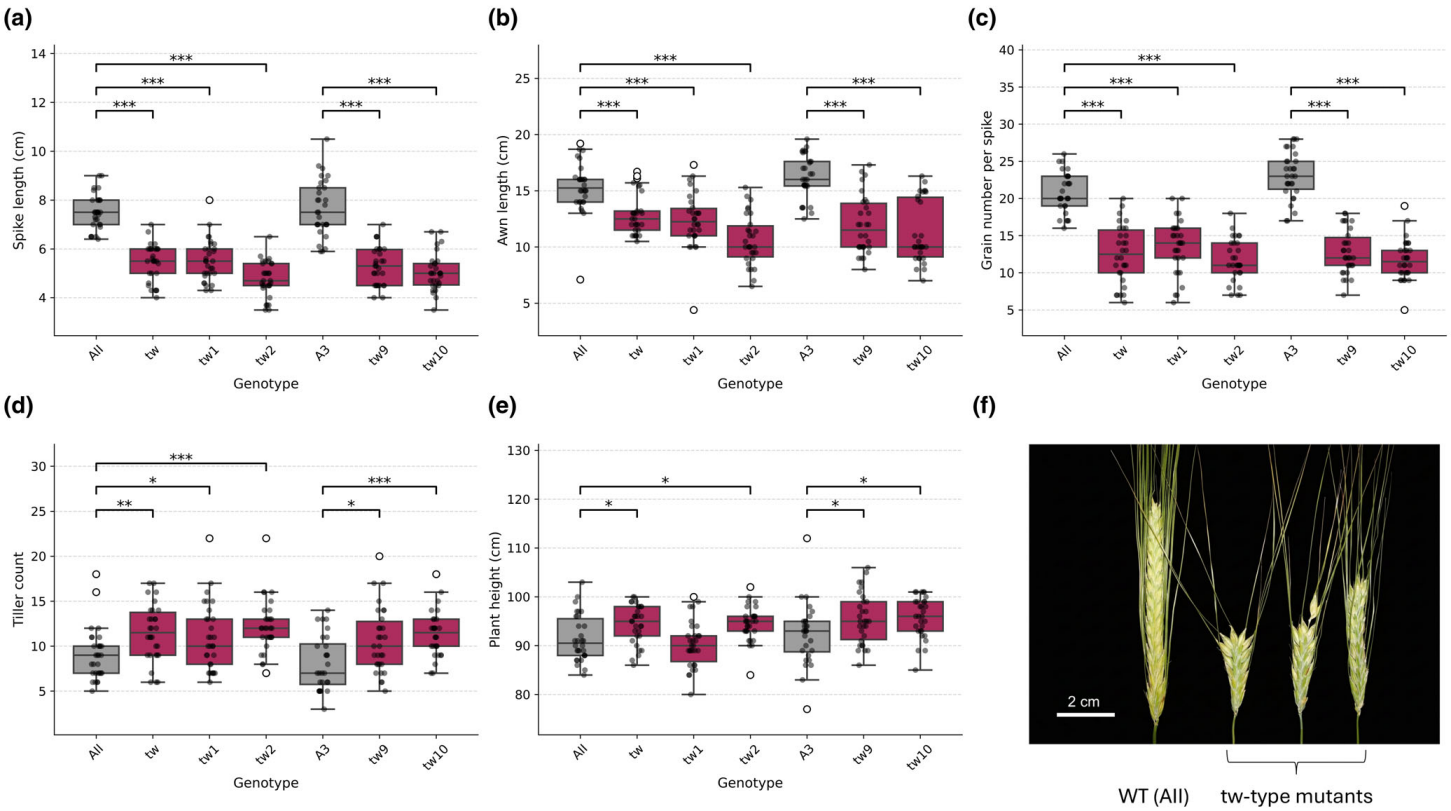
Morphometric traits of *tweaky spike* (*tw*) barley (*Hordeum vulgare* L.) mutants and their respective wild‐type (WT) backgrounds, evaluated over three years (2021–2023). Analyzed traits include (a) spike length (cm), (b) awn length (cm), (c) grain number per spike, (d) tiller count per plant, and (e) plant height (cm). (f) Features of WT and *tw*‐type mutant spikes. Data are presented as boxplots (gray color – WT (AII and A3), pink color – *tw*‐type mutants) showing median, interquartile range, and individual data points represented as translucent black circles, outliers as white circles (*n* = 30, except for A3 plant height and tiller count *n* = 28). WT controls: AII for *tw*, *tw1*, and *tw2*; A3 for *tw9* and *tw10*. Differences were assessed using the Mann–Whitney U test, followed by the Benjamini–Hochberg correction for multiple comparisons. Asterisks indicate significant differences compared to the corresponding WT: *, *P* < 0.05; **, *P* < 0.01; ***, *P* < 0.001.

Across all allelic mutants, a consistent reduction in spike length and awn length was observed (Fig. 1a,b, *P* < 0.001), which strongly correlated with a decreased grain count per spike compared to their WT counterparts (*P* < 0.001, Pearson *r* = 0.83, Fig. S1a). Moreover, *tw* mutants exhibited more sterile spikelets per spike (*P* < 0.05, Fig. S1b), indicating compromised spike fertility and development.

*tw* mutants tended to be taller (Fig. 1e, *P* < 0.05, except for *tw1*) and produced more tillers per plant (Fig. 1d, *P* < 0.01) than their respective WT backgrounds. Although these differences were less robust, the consistent trends indicate that the mutation also affects vegetative development, not only reproductive traits.

### *tw* mutation effect on barley inflorescence

The *tw* mutation in barley leads to a range of inflorescence abnormalities, affecting both spike architecture and floral organ development. Over the years, several distinct phenotypes were consistently observed in *tw* allelic mutants, including lodicule transformations, deviations in the number of floral organs, sterile spike base, spikeletless rachis node/elongated internode (spike gap), paired spikelets on the top of the spike, and an overdeveloped terminal part referred to as a crowned spike tip (Fig. 2a–d).

**Fig. 2:**
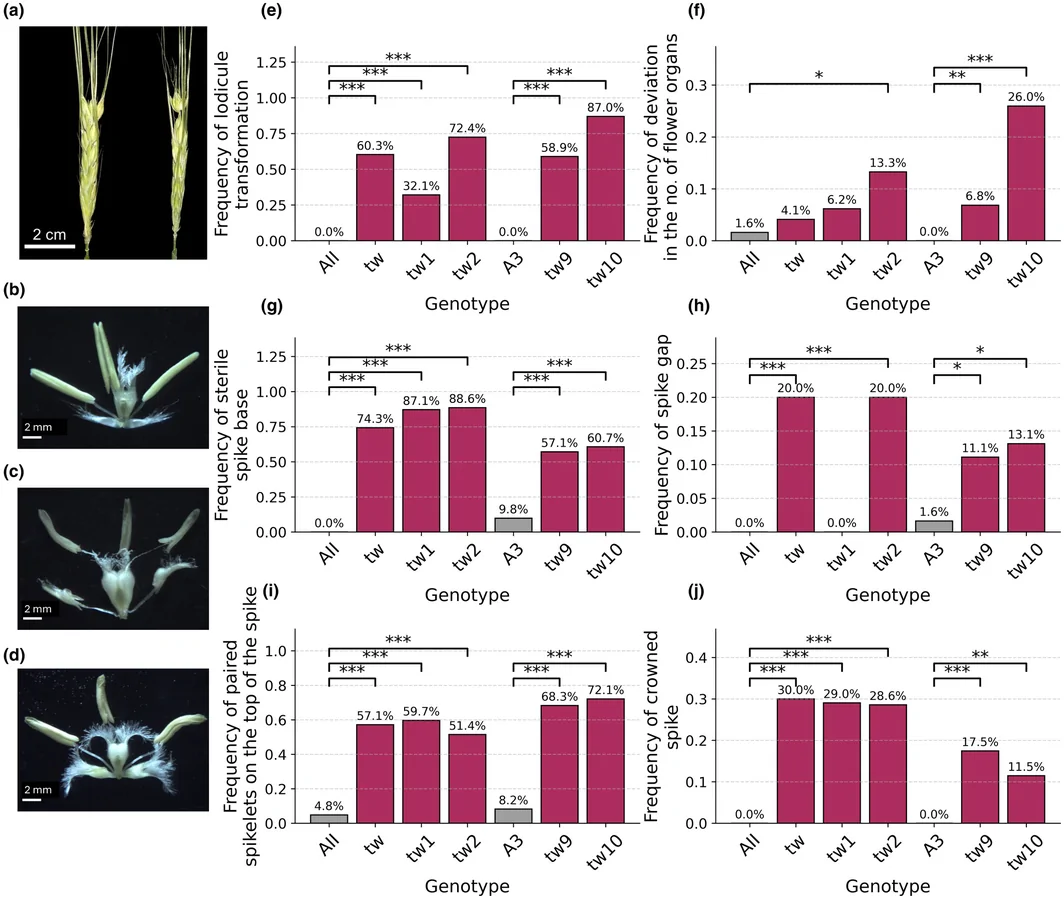
Inflorescence/floret phenotypes of *tweaky spike* (*tw*) barley (*Hordeum vulgare* L.) mutants and their respective wild‐type (WT) backgrounds. (a) *tw*‐type mutants spikes: (left: spike with crown, right: spike with crown and spike gap) (b) WT barley floral organs, (c) lodicule transformation to stamen, (d) lodicule transformation to pistil. Measured traits include (e) frequency of lodicule to reproductive organ transformation, (f) frequency of deviation in the number of floral organs, (g) frequency of sterile spike base, (i) frequency of spikeletless rachis node/elongated internode (spike gap), (j) frequency of presence of two spikelets at the top of the spike, and (k) frequency of presence of a crowned spike tip. Data are presented as bar plots showing the proportion of spikes exhibiting each phenotype (*n* > 60, exact value in Supporting Information Tables S5 and S6). WT controls: AII for *tw*, *tw1*, and *tw2*; A3 for *tw9* and *tw10*. Differences in phenotype frequencies were assessed using a two‐proportion Z‐test. Asterisks indicate significant differences compared to the corresponding WT: *, *P* < 0.05; **, *P* < 0.01; ***, *P* < 0.001.

These traits were recorded as present or absent in individual spikes or spikelets, then quantified by scoring the proportion of spikes/spikelets expressing each phenotype (Fig. 2e–k; Tables S5 and S6). The analysis revealed that expressivity varied significantly across allelic mutants, despite all being nonfunctional variants of the same gene (p values of all comparisons listed in Table S7). For example, *tw1* exhibited significantly fewer lodicule transformations and no detectable spike breaks, whereas *tw10* showed the highest deviation in floral organ number among all allelic lines. Although some of these abnormalities occasionally appeared in WT plants (AII or A3), they were rare, which further supports that the observed phenotypes in *tw* lines are mutation‐driven.

The expressivity of certain traits also differed between mutants originating from distinct WT backgrounds (AII for *tw*, *tw1*, and *tw2*, and A3 for *tw9* and *tw10*) (Table S7), suggesting that genetic background (either from different WT or secondary mutations from independent mutagenesis) modulates the penetrance or expressivity of the tw phenotype. Such differences point toward possible gene‐by‐background interactions that influence inflorescence development.

Taken together, these findings demonstrate that the *tw* mutation not only disrupts the organization and fertility of the barley spike but also causes variable effects on floral organ identity and meristem determinacy, depending on the allelic variant and its genetic context.

### A mutation in the *HvAP2L5* gene results in a tweaky spike phenotype

To identify the genomic locus associated with the tw phenotype, we applied the MutMap approach (Abe *et al*., 2012). Two allelic mutants, *tw* and *tw2*, were outcrossed to their corresponding WT background cultivar ‘Auksiniai II’ (AII). The F_1_ plants were phenotypically WT and self‐pollinated to generate F_2_ populations. Segregation of the mutant phenotype in the F_2_ generation was consistent with a monogenic recessive inheritance pattern (*tw‐F*_*2*_: 1588 WT, 517 mutants, χ^2^ = 0.22; *tw2‐F*_*2*_: 943 WT, 326 mutants, χ^2^ = 0.32).

SNP‐ratio analysis revealed two distinct non‐recombinant peaks associated with the tw phenotype: one on Chromosome 1 (1H: 3.2–6.8 Mb) and another on Chromosome 5 (5H: 530–580 Mb) (Figs 3a, S2, S3). These regions represent the primary candidate intervals for the *tw* locus.

**Fig. 3:**
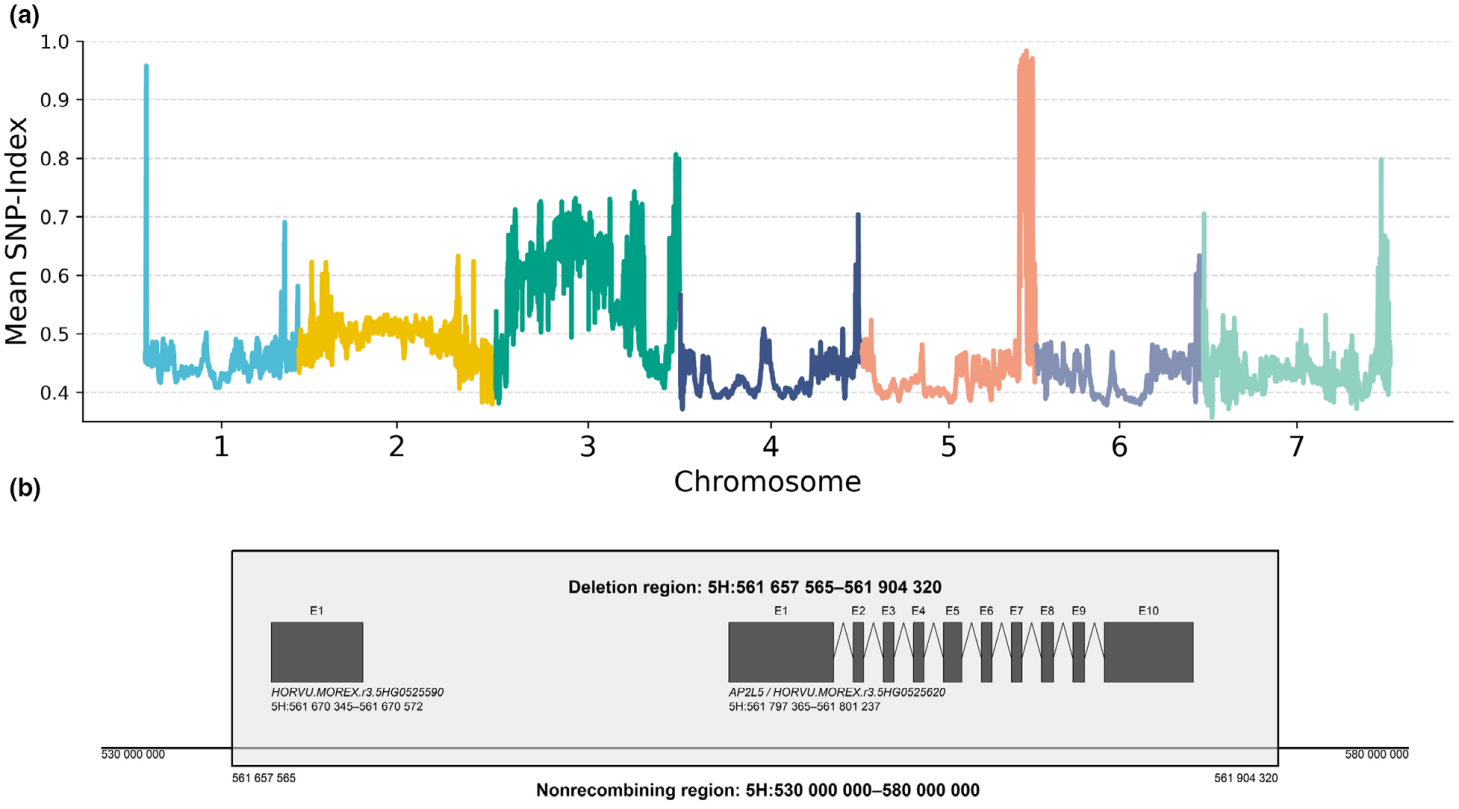
Nonrecombining region and deletion locations in barley (*Hordeum vulgare* L.) *tw‐F*_*2*_ and *tw2‐F*_*2*_ populations. (a) Genome‐wide single‐nucleotide polymorphism (SNP)‐index (rolling mean in 500‐SNP windows) distribution of the *tw2‐F*_*2*_ mutant bulk across the seven barley chromosomes. (b) Genomic locations of the deletions identified in the *tw* and *tw2* mutants. The deletion predicted by dysgu spans chromosome 5H from 561 657 565 to 561 904 320 bp within the mapped nonrecombining interval of 530–580 Mb. The deletion encompasses *HvAP2L5* (*HORVU.MOREX.r3.5HG0525620*) and the adjacent gene *HORVU.MOREX.r3.5HG0525590*. Exons are represented by gray boxes, and introns are shown as connecting lines. Genomic coordinates are based on the MorexV3 reference genome.

All SNPs identified in the nonrecombinant regions (1H: 3.2–6.8 Mb and 5H: 530–580 Mb) were annotated and analyzed further. Filtered SNPs from *tw‐F*_*2*_ and *tw2‐F*_*2*_ were then compared to the AII pool, and any variants present in the WT pool (even at low frequency) were removed. After filtering, no candidate genes remained in the 1H: 3.2–6.8 Mb interval, and this region was therefore discarded as not associated with the tw phenotype. By contrast, the 5H: 530–580 Mb region contained 25 candidate genes with missense mutations in *tw* and 63 in *tw2*. Given that *tw* and *tw2* are allelic, the causal gene should be disrupted in both mutants. Only five disrupted genes were shared between the two datasets. Among these, two genes harbored SNPs already annotated in the Morex V3 genome and present in WT populations, and the remaining three contained SNPs predicted as tolerated by SIFT. These results indicated that the SNP‐based approach could not fully explain the tw phenotype.

To investigate structural variants, we employed the dysgu (Cleal & Baird, 2022) program. A deletion within the nonrecombining region was identified in both mutants and contained two genes (Fig. 3b). *HORVU.MOREX.r3.5HG0525590* was excluded as a candidate because the Morex reference genome contains an annotated frameshift polymorphism in this gene, indicating that its disruption is unlikely to be associated with the tw phenotype. This left *HORVU.MOREX.r3.5HG0525620* as the single strong candidate (WGS coverage across the *HvAP2L5* locus provided in Fig. S4). To validate this gene as the causal locus, we designed primers (primers list: Table S1, primer placement: Fig. S5) and analyzed its sequence in additional independently derived *tw* allelic mutants (*tw1*, *tw4*, *tw5*, *tw6*, *tw8*, *tw9*, and *tw10*). PCR analysis (Fig. S6) revealed that all mutants carried a full gene deletion, except for *tw1*. Sanger sequencing analysis revealed that the ORF in *tw1* was intact and carried no deleterious SNPs (cDNA is identical between AII and *tw1*; Fig. S7). Comparison of the *HvAP2L5* upstream region among *tw1* and the two WT genotypes, (AII and A3), revealed a 62‐nt deletion in *tw1* immediately upstream of the 5′ UTR (five prime untranslated region) that was absent from both WT (Fig. S8). To test whether a regulatory mutation caused the tw phenotype in the *tw1* mutant, we examined *HORVU.MOREX.r3.5HG0525620* expression in two developmental stages. Transcripts of the target gene were detected in AII WT (Ct = 25), but no expression was detected in *tw1* (Fig. S9) at the coleoptile stage, suggesting transcriptional silencing due to a regulatory defect. However, *HORVU.MOREX.r3.5HG0525620* expressed at low levels in developing spikes of *tw1* although expression remained significantly lower than in the WT (*tw1* Ct = 36 and WT Ct = 25) (Fig. S10).

Collectively, these findings demonstrate that *HORVU.MOREX.r3.5HG0525620*, also known as *HvAP2L5*, is disrupted in all *tw* allelic mutants, either through complete gene deletion or through regulatory silencing, and is therefore the gene responsible for the *tw* phenotype in barley.

We also validated gene function by creating the *HvAP2L5* gene knockout in cv ‘Golden promise’ using the CRISPR‐Cas9 system. We generated 17 T0 plants carrying T‐DNA insertions, one of which (*tw.c1*) displayed a 490–777 bp inversion between Cas9 targets T3 and T4 (Fig. S11). *tw.c1* possessed a *tw* phenotype, forming crowned spike tips (Fig. S12; Table S8) and lodicule transformations (Table S9), confirming *HvAP2L5* function in flower/inflorescence development.

### *HvAP2L5* gene expression levels in different development stages

*HvAP2L5* was transcriptionally active across all examined stages (Fig. 4a). Expression peaked in coleoptiles (average relative expression (normalized to housekeeping genes) was 0.954) and at the 5th leaf stage (average relative expression was 0.879), indicating strong activity during early seedling growth. Significantly lower expression levels were observed in roots compared to 5th leaf stage samples (*P* < 0.05, average relative expression was 0.371). Lower expression was also observed in developing spikes (average relative expression was 0.518), although this difference was not statistically significant. These results suggest that elevated *HvAP2L5* expression at the 5th leaf stage, when inflorescence initiation begins, together with its continued expression during spike development, may coordinate developmental processes involved in subsequent inflorescence formation.

**Fig. 4:**
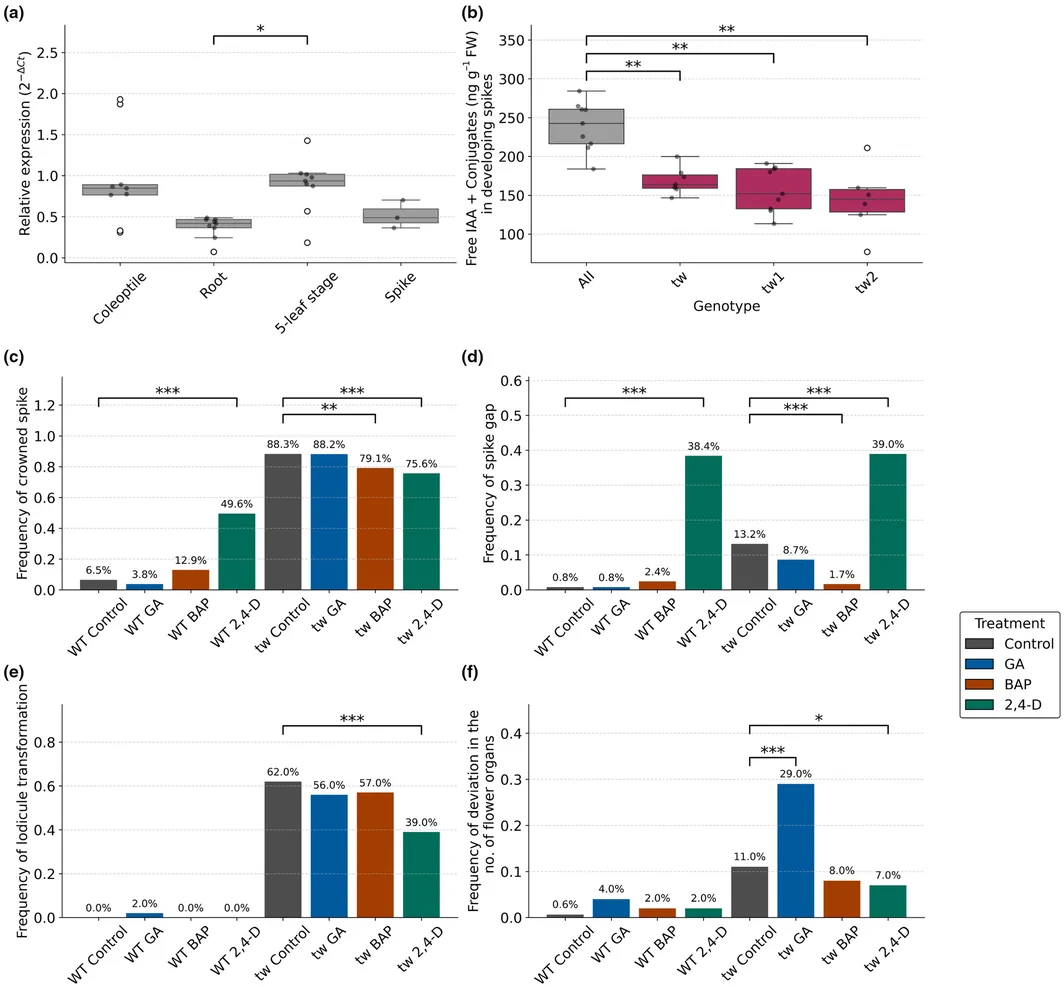
Phytohormones association with *tweaky spike* (tw) phenotype. (a) Relative expression of the *HvAP2L5* gene in different barley (*Hordeum vulgare* L.) development stages (coleoptile, root, 5‐leaf stage leaf and developing spike), data are presented as boxplots showing median, interquartile range, and individual data points represented as translucent black circles, outliers as white circles. (b) Endogenous auxin (IAA) and its conjugates levels in *tw*‐type mutants and wild‐type (WTs) developing spikes, data are presented as boxplots (gray color – WT (AII), pink color – *tw*‐type mutants) showing median, interquartile range, and individual data points represented as translucent black circles, outliers as white circles. Exogenously applied phytohormones (auxin: 2,4‐D, gibberellin: GA, cytokinin: BAP) effect on phenotypes associated with *tw*‐type mutants: (c) frequency of crowned spikes, (d) frequency of spike gap, (e) frequency of lodicule transformation, (f) frequency of deviation in the number of flower organs. Differences in relative expression and IAA levels were assessed using the Mann–Whitney U test, followed by the Benjamini–Hochberg correction for multiple comparisons (a, b). Differences in phenotype frequencies were assessed using a two‐proportion Z‐test (c–f). Asterisks indicate significant differences compared to the corresponding WT: *, *P* < 0.05; **, *P* < 0.01; ***, *P* < 0.001.

### Phytohormones influence on flower and spikelet development

Among the exogenously applied phytohormones, only synthetic auxin (2,4‐D) had a consistent and statistically significant impact on all four tested *tw*‐associated traits (Fig. 4c‐f). In WT plants, auxin treatment induced the formation of crowns and spike gaps, phenotypes usually absent in untreated WT, which suggests that elevated auxin levels alone can partially reproduce the phenocopies of *tw* characteristics. Conversely, in *tw* mutants, auxin treatment led to a notable reduction in the frequency of crown formation, lodicule transformation, and floral organ number variation, implying a partial phenotype rescue. This suggests that *tw* mutants may have impaired auxin biosynthesis, signaling, or distribution, which can be partially compensated by external auxin application.

GA and CK treatments influenced specific inflorescence and/or floral traits but lacked consistent effects across all tested features (Fig. 4c–f). GA3 increased the frequency of deviations in floral organ number in *tw* plants but had no effect on other features or on WT plants (Fig. 4f). CK treatment reduced the frequency of spike gaps and crowns in *tw*, while WT remained largely unaffected (Fig. 4c,d). These results imply that the primary hormonal pathway affected by the *tw* mutation is related to auxin regulation. By contrast, the effects of GA and CK are likely secondary or modulated by crosstalk with auxin pathways.

To verify the hypothesis of disrupted auxin homeostasis in *tw* mutants, endogenous auxin (IAA) levels were quantified in coleoptile (Fig. S13) and developing spike (Fig. 4b) tissue of *tw* mutants and WT lines. All tested allelic *tw* mutants consistently exhibited significantly lower total IAA concentrations (free and conjugated IAA) than WT plants, confirming a biochemical basis for the impaired auxin signaling in mutants.

## Discussion

Mutant research, whether generated by random mutagenesis (e.g. irradiation, chemical treatments) or by targeted approaches such as CRISPR, remains a cornerstone for elucidating gene function in barley and other cereals (Lundqvist & Franckowiak, 2003; Harwood Editor, 2019; Hansson *et al*., 2024). Since the late nineteenth century, the widespread use of mutagenesis for trait discovery has generated vast mutant collections that often lack immediate agronomic value but are interesting for gene function analysis (Terzi *et al*., 2017; Hansson *et al*., 2024). Because each laboratory historically assigned its own nomenclature to newly discovered phenotypes, the same underlying gene is frequently represented by multiple, sometimes conflicting, mutant names and descriptions (Hansson *et al*., 2024). This lack of uniformity leads to duplicated effort, as researchers may unknowingly study identical loci under different labels. Yet, it also offers the benefit that independent investigations can reveal distinct aspects of gene function and uncover novel phenotypic nuances. Consequently, the barley genetics community faces a paradoxical situation: the chaotic proliferation of mutant names hampers efficient knowledge integration while simultaneously providing diverse perspectives that can accelerate discovery.

### The *tweaky spike* phenotype is caused by a loss‐of‐function mutation in *the HvAP2L5* gene

A series of unique barley mutants with disturbed inflorescence development was created in the Vilnius University Department of Botany and Genetics. These mutants were called *tw*, according to Agriculture handbook No. 338 (Science and Education Administration 1979). The mutants feature crowned spike tips, spikeletless rachis nodes/elongated internodes (spike gaps), paired opposite spikelets at the apex, sterile spike bases, lodicule transformations, and an irregular number of floral organs. The spike and awns are shorter, and grain number per spike is reduced (Figs 1, 2; Fig. S1). Some allelic lines also showed a tendency toward increased plant height and tiller number (Fig. 1d,e). Genetic mapping revealed that the gene responsible for this phenotype is *HORVU.MOREX.r3.5HG0525620*, also known as *HvAP2L5*. There are two other known mutant lines with the disrupted *HvAP2L5* gene – *intermedium‐m* (*int‐m*) and *double seed1* (*dub1*). The *int‐m* and *dub1* mutants convert normally indeterminate barley spike into a determinate: inflorescence terminating in a multifloreted terminal spikelet (Zhong *et al*., 2021). The same terminal structure is observed in the *tw* mutants and is referred in our study as the crown phenotype because of its characteristic crown‐like appearance at the spike apex. Thus, the crown phenotype described in this study corresponds to the multifloreted terminal spikelet phenotype reported by Zhong *et al*. (2021). This conversion reduces the total number of spikelets and produces spikelets with extra florets, which are often sterile, due to desuppressed residual spikelet meristems (Zhong *et al*., 2021). Importantly, our analysis showed that the crown phenotype was not present in all spikes but occurred at variable frequencies among the *tw* alleles, demonstrating variation in the expressivity of the terminal spikelet phenotype (Fig. 2).

### *HvAP2L5* controls lodicule identity

Our study sheds new insights into the phenotype caused by a loss‐of‐function mutation in the *HvAP2L5* gene. We discovered that *HvAP2L5* affects not only inflorescence development but also floret development. When *HvAP2L5* function is impaired, lodicule differentiation is disrupted, with lodicules converting into other floral organs such as stamens or pistils (Fig. 2). Consistent with this, the frequency of lodicule‐to‐stamen/pistil conversion is significantly elevated in the *tw* alleles than in WT (up to *c.* 0.87 vs 0), demonstrating that *HvAP2L5* is essential for maintaining lodicule identity.

*HvAP2L5* belongs to the A‐class of floral‐organ‐identity genes, which specify the first two whorls of the flower: the outer bract‐like whorl (lemma‐palea) is determined solely by A‐class activity, whereas the second whorl (lodicules in grasses) requires combined A‐ + B‐class function (Krogan *et al*., 2012; Callens *et al*., 2018; Kuijer *et al*., 2021). In A‐class mutants, carpel‐like transformations occur in the first and second whorls (Coen & Meyerowitz, 1991; Causier *et al*., 2010), exactly the phenotype observed in *tw/hvap2l5* barley mutants, where lodicules are frequently transformed into stamen‐ or pistil‐like structures (Fig. 2). *HvAP2L5* expression was also detected in lemmas and paleas (Zhong *et al*., 2021), together with lodicule transformations showing gene involvement in the first two whorls of the flower development in barley.

Moreover, A‐class activity is driven by a small gene family rather than a single locus. Consequently, a mutation in one A‐class gene does not necessarily eliminate first‐ and second‐whorl identity. Instead, partial redundancy among paralogous A‐class genes can maintain organ‐identity specification, preserving elements of floral organ identity when one determinant is compromised (Wellmer *et al*., 2006; Causier *et al*., 2010). We therefore propose that the incomplete penetrance and variable expressivity of lodicule transformations in *tw/hvap2l5* mutants reflect compensatory activity from other A‐class factors that maintain floral whorl identity to some extent, but not at WT levels.

Parallel evidence for A‐class redundancy comes from other *AP2‐*like genes in cereals. In barley, loss‐of‐function alleles of the closely related *HvAP2L2* (*gigas1.a* and *hvap2‐2*) produce enlarged, swollen lodicules that can develop glume‐like hairs and, at low frequency, undergo partial conversion into hybrid stamen/bract‐like organs (Shoesmith *et al*., 2021). These abnormalities resemble key aspects of the *tw/ap2l5* mutant phenotype, supporting overlapping roles of *HvAP2L2* and *HvAP2L5* in lodicule fate determination. Conversely, the *HvAP2L2* gain‐of‐function *Zeo1.b* and *Cly1* alleles drive the opposite outcome: lodicules remain small, fail to swell at anthesis, and florets remain closed, conferring cleistogamy (Nair *et al*., 2010; Houston *et al*., 2013). Wheat *AP2* class genes also support the idea of partial redundancy: while *ap2l5* mutants show lodicule transformation at a lower frequency than in barley, *ap2l2ap2l5* double mutants display complete loss of lodicules, indicating that these paralogs act jointly to specify lodicule determinacy (Debernardi *et al*., 2020).

Taken together, these observations are fully consistent with the canonical ABC model of flower development, while emphasizing that grasses have expanded A‐class functional redundancy. The strong parallels between barley and wheat further indicate that *Apetala2‐like (AP2L)* clade activities are highly conserved within the tribe of Triticeae. Beyond this group, related regulatory roles have been documented for the rice *AP2L* orthologs *SUPERNUMERARY BRACT* and *INDETERMINATE SPIKELET1* (*OsIDS1*), which influence inflorescence architecture and floral meristem identity (Lee *et al*., 2007; Lee & An, 2012). Considered alongside the well‐established functions of *AP2* in Arabidopsis, including perianth identity, seed development, and meristem longevity, these data further underscore that *AP2L* genes form an ancient developmental toolkit (Zumajo‐Cardona *et al*., 2021). Accordingly, our findings position *HvAP2L5* within a broadly conserved regulatory network that has been repeatedly adapted during grass evolution and domestication to coordinate inflorescence determinacy, floret formation, and lodicule fate.

Controversy, *tw/ap2l5* mutants exhibit deviation in the number of other floral organs (Fig. 2). In WT barley, each floret typically contains two lodicules, three stamens, and one pistil. By contrast, *tw* mutants display not only lodicule transformations but also frequent changes in stamen and pistil number: pistils can range from 0 to 2 and stamens from 1 to 5. This variability suggests that *HvAP2L5* influences not only lodicule identity but may also affect patterning of the third and fourth whorls. While the antagonistic A‐C gene separation is a core of the ABC model in Arabidopsis (Krogan *et al*., 2012), barley C‐class genes (*HvMADS*3 and *HvMADS58*) unexpectedly expressed in second‐whorl lodicules, breaking this spatial separation. This suggests grasses tolerate overlapping expression domains that eudicots suppress (Kuijer *et al*., 2021).

The increased frequency of sterile basal spikelets in the *tw* mutants further indicates that *HvAP2L5* is required for normal floret development throughout the spike. A comparable basal phenotype has been reported in wheat *AP2L5/Q* loss‐of‐function mutants, which produce rudimentary basal spikelets and empty lemmas lacking associated floral organs (Debernardi *et al*., 2017). This similarity suggests that the role of *AP2L5* in promoting basal spikelet fertility and floral meristem development is conserved within the Triticeae.

### *HvAP2L5* is expressed in vegetative organs and suppresses axillary meristems in barley

Our results indicate that *HvAP2L5* gene activity extends beyond the previously characterized reproductive stages, showing significant expression during early vegetative growth, including roots, coleoptile, and 5‐leaf stage (Fig. 4a). This vegetative activity is consistent with the expression profiles of *AP2L5* homologs in wheat, which are detected in vegetative apices before the reproductive transition. *AP2L5* expression levels in wheat remain relatively constant from the vegetative apex through the floret primordia stage (Simons *et al*., 2006; Gil‐Humanes *et al*., 2009; Debernardi *et al*., 2020). Given its presence in these early stages, loss‐of‐function mutations in *ap2l5* would be expected to alter vegetative shoot architecture. The observation that all *tw* exhibit increased tiller numbers suggests that *HvAP2L5* functions as a negative regulator of axillary meristem (AM) initiation or outgrowth during the vegetative phase. Consistent with a broader developmental role, altered regulation of wheat *AP2L5*, including miR172‐resistant *AP2L‐A5* alleles, has been associated with reduced plant height (Debernardi *et al*., 2019). Together, these observations support the hypothesis that *AP2L5* genes regulate not only floral and inflorescence development but also vegetative growth traits.

### *HvAP2L5* association with phytohormones

Our findings show that *tw* mutants have reduced endogenous auxin levels in both coleoptiles and developing spikes. At the coleoptile stage, all tested allelic mutants (*tw*, *tw1*, *tw2*, *tw9*, and *tw10*) exhibited lower IAA concentrations than their corresponding WTs, while developing spikes of *tw*, *tw1*, and *tw2* similarly showed reduced levels of free IAA and its conjugates (Figs 4b, S13). These consistent reductions across vegetative and reproductive tissues may provide a biochemical basis for the observed rescue effect of synthetic auxin. The application of 2,4‐D during early development likely compensates for this deficiency, reducing the frequency of abnormal spike features such as spikeletless gaps, crown formation, and floral organ transformations (Fig. 4) (Šiukšta *et al*., 2018). In WT barley, regular spike patterning and phase duration are maintained by sophisticated inverse basal‐apical gradients of auxin and cytokinin (Youssef *et al*., 2017; Youssef & Hansson, 2019). The mutation in the *HvAP2L5* gene may disrupt this delicate hormonal homeostasis, leading to an uneven distribution of auxin and other phytohormones across separate spike phytomers. Our qPCR analysis identified changes in the expression of several genes involved in auxin transport and signaling in developing *tw* spikes (Fig. S4). Reduced expression of *AUX1/LAX1* and *LAX2/LAX3* was observed in *tw1*, while *ARF3b* expression was increased in the *tw*‐type mutants. Although these findings partially overlap with the auxin‐related transcriptional changes reported by Zhong *et al*. (2021), the overall expression patterns were not fully consistent and may depend on the mutant allele, genetic background, and developmental stage.

Interestingly, our results demonstrate that the characteristic tweaky phenotype can be induced by both an endogenous auxin deficiency (as seen in the *tw* mutants) and an induced auxin excess (as observed in WT plants after 2,4‐D treatment) (Fig. 4) (Šiukšta *et al*., 2018). Previous experiments also showed that the polar auxin transport inhibitor 9‐hydroxyfluorene‐9‐carboxylic acid (HFCA) and the anti‐auxin p‐chlorophenoxyisobutyric acid (PCIB) partially normalized several *tw2* floral abnormalities, including lodicule transformations, although these effects were genotype‐ and trait‐dependent (Šiukšta *et al*., 2015). Together, these findings suggest that proper spike differentiation depends not only on maintaining auxin concentration within an optimal range but also on preserving auxin homeostasis, spatial distribution, and cellular responsiveness within developing spike tissues. Disturbance in auxin abundance, transport, or signaling may therefore disrupt the initiation and arrangement of new spikelet primordia at the shoot apex.

The significant impact of auxin compared to the mild, specific effects of other phytohormones (gibberellin, cytokinin; Fig. 4) suggests that the latter are likely side effects of a collapsed auxin gradient (Youssef & Hansson, 2019; Voytenko & Kosakivska, 2025). Consequently, loss‐of‐function mutation in the *HvAP2L5* gene primarily triggers a fundamental disruption in auxin physiology, which subsequently destabilizes the broader hormonal crosstalk required for precise floral organ specification and spikelet development. This collapse of coordinated hormonal gradients may provide a mechanistic explanation for the genetic instability and wide spectrum of morphological variations observed in the *tw* mutant spikes (Šiukšta *et al*., 2018).

Furthermore, most of the *tw* phenotypic changes can be linked to auxin deficiency. In Poaceae, higher auxin levels are associated with increased spike size (Ali *et al*., 2009; Feng *et al*., 2023) and awn length (Qiao *et al*., 2025) as well as grain size/yield (Liu *et al*., 2015; Shao *et al*., 2017); therefore, the reduced expression of these features may result from lower internal IAA concentration in *tw* mutants. *tw* mutants also exhibit a higher proportion of *Fusarium* spp. in the internal fungal community (Šiukšta *et al*., 2021). This susceptibility may also be linked to auxin deficiency, as auxin participates in cereals' responses to major fungal pathogens such as *Fusarium* (Petti *et al*., 2012; Mapuranga *et al*., 2026).

Comparative studies suggest that the monocot lodicule is structurally homologous to the dicot petal, consistent with a shared evolutionary origin followed by divergence in form and function (Nair *et al*., 2010). Notably, auxin perturbation can drive organ‐identity shifts in petal‐derived tissues: altered auxin levels induce homeotic transformations between petals and stamens in rose (*Rosa hybrida*) (Chen *et al*., 2021). These findings support the idea that the lodicule transformations observed in *tw* mutants could be linked to defects in auxin physiology. In line with this, the AP2 transcription factor has been described as a master regulator of auxin homeostasis, influencing auxin biosynthesis, conjugation, transport, and signaling: loss of AP2 function in woodland strawberry disrupts auxin distribution and availability and is accompanied by developmental abnormalities, including altered floral organ formation (Lu *et al*., 2024). Together, these studies, lower auxin concentrations in *tw* mutants and exogenous auxin application rescue effect provide a mechanistic rationale to connect *AP2/AP2*‐like mutations with auxin imbalance and to implicate disrupted auxin regulation as a plausible driver of lodicule fate alterations in *tw*.

### *Tweaky spike* phenotype instability

The tw phenotype exhibits significant instability and differential expressivity, likely modulated by the genetic background and the presence of modifier mutations. Even independently derived allelic mutants sharing the same parental background (cv ‘Auksiniai II’ or cv ‘Auksiniai 3’) showed significant differences in the frequency of specific inflorescence abnormalities (Table S7). For instance, *tw10* (A3 background) displayed the highest deviation in floral organ number among all lines, while *tw9* (also A3 background) had five times lower frequency of the same trait. Such differences point toward complex gene‐by‐background interactions that influence inflorescence development.

Furthermore, the nature of the genetic lesion in the *HvAP2L5* (*HORVU.MOREX.r3.5HG0525620*) locus appears to influence the tw phenotype expressivity. While most analyzed alleles (*tw*, *tw2*, *tw4–tw10*) carry a complete ORF deletion, *tw1* retains an intact gene sequence (Fig. S7). *HvAP2L5* in *tw1* is transcriptionally silenced in the coleoptile stage, but a low expression was detected in developing spikes (Figs S9 and S10). This transcriptional downregulation might be caused by a 62 nt deletion in the *tw1* upstream region (Fig. S8). Interestingly, *tw1* exhibited the lowest frequency of lodicule transformations and no detectable spike gaps (Fig. 2), PCA from plant and spike morphometic data placed the *tw1* mutant closest to WT compared to other allelic *tw* mutants (Fig. S15). We hypothesize that minimal expression during early spike development may contribute to the comparatively milder *tw1* phenotype relative to the null alleles.

Despite these variations in penetrance and expressivity, the *tw* mutation remains fundamentally stable in its core manifestations. Four definitive traits, the formation of crowned spike tips, a sterile spike base, lodicule transformations, and floral organ number deviations, were consistently observed across all tested allelic lines. These features define the robust core of the tw phenotype, persisting regardless of the specific genetic background or allelic variant. The presence of these hallmark characteristics in all independent mutants demonstrates that while genetic context modifies the intensity of the phenotype, the underlying developmental disruption driven by the *HvAP2L5* lesion is consistent.

### Conclusion

The barley *tw* allelic series represents a distinctive set of pleiotropic inflorescence and floret mutants, consistently displaying shortened spikes with basal sterility, apical crown formation, and unstable lodicule‐to‐reproductive organ transformations. By combining genetic mapping, structural variant analyses, and functional validation, we demonstrate that loss of *HvAP2L5* function (via full ORF deletion in most alleles or transcriptional silencing in *tw1*) causes the tw phenotype in barley. Importantly, *tw* mutants exhibit a reproducible endogenous auxin (IAA) deficit. By contrast, exogenous auxin can partially phenocopy or rescue key traits, indicating that disrupted auxin homeostasis might be the key element in tw phenotype expressivity. We propose that auxin deficiency and resulting hormonal imbalance provide a unifying mechanistic explanation for the majority of *tw* pleiotropic features.

## Competing interests

None declared.

## Author contributions

VP and RŠ conceived and designed the study. VP and LT constructed vectors. VP and MR performed barley transformation and regeneration. MR did gene validation using PCR. BKM did expression analysis and interpretation. AL collected morphometric data. VV did barley hybridization experiments. Jurga Jankauskienė, SJ and JS did auxin concentration analysis. Julius Juodakis and VP did bioinformatic analysis. VP and RŠ interpreted the data. VP wrote the manuscript. VP, BKM, MR, Jurga Jankauskienė, Julius Juodakis, SJ, AL, LT, JS and RŠ revised the manuscript.

## Disclaimer

The New Phytologist Foundation remains neutral with regard to jurisdictional claims in maps and in any institutional affiliations.

## Supporting information


**Dataset S1** Morphometric measurements of barley (*Hordeum vulgare*) genotypes used in this study. The dataset contains individual measurements of spike length, awn length, sterile spikelet number, grain number, plant height, and tiller number.

**Fig. S1** Agronomic traits of *tweaky spike* (*tw*) barley mutants and their respective wild‐type backgrounds, evaluated over three years (2021–2023).
**Fig. S2** Genome‐wide distribution of the SNP‐index in the *tw‐F2* mutant bulk.
**Fig. S3** Genome‐wide distribution of the SNP‐index in the *tw2‐F2* mutant bulk.
**Fig. S4** Sequencing coverage across the *HvAP2L5* locus in the wild‐type and mutant samples.
**Fig. S5** Annotated genomic sequence of *HvAP2L5* (*HORVU.MOREX.r3.5HG0525620*) from Morex.
**Fig. S6** Amplification of fragment W4 from genomic DNA using primer pair AP2L5/W4.
**Fig. S7** ‘Auksiniai II’ (AII) and *tw1 HvAP2L5* coding sequence alignment (*HORVU.MOREX.r3.5HG0525620*) location: 5H: 561797365‐561 801 237.
**Fig. S8** Sequence alignment of the *HvAP2L5* gene (*HORVU.MOREX.r3.5HG0525620*) upstream region from ‘Auksiniai II’, ‘Auksiniai 3’, and *tw1*.
**Fig. S9** Log‐scaled RT‐qPCR amplification curves of the target gene *HvAP2L5* (HORVU.MOREX.r3.5HG0525620) and the reference genes *HvADP* (*HORVU.MOREX.r3.6HG0606370*) and *Snor14* (*LOC123445868*) in coleoptile tissue of wild‐type ‘Auksiniai II’ and *tw1* mutant plants.
**Fig. S10** Relative expression of *HvAP2L5* in developing spikes of the wild‐type (AII) and *tw1* genotypes.
**Fig. S11** Sequence analysis of *tw.c1* CRISPR‐Cas9 allele.
**Fig. S12** Representative photos of 75‐d old spike from wild‐type and *tw.c1* plants.
**Fig. S13** Free IAA and IAA conjugate levels in coleoptiles of *tw* mutants and their corresponding Wild‐Type genotypes, determined using high‐performance thin‐layer chromatography.
**Fig. S14** Expression of genes involved in auxin transport, signaling and homeostasis in developing spikes of AII, *tw* and *tw1* genotypes.
**Fig. S15** PCA created from agronomic and spike/flower morphometric parameters.
**Table S1** Oligonucleotides used for amplification of *HvAP2L5* gene fragments and single‐guide RNAs used for CRISPR/Cas9‐mediated editing of *HvAP2L5*.
**Table S2** Primers used for qPCR analyses.
**Table S3** Morphometric traits of tw barley mutants and Wild‐Types: mean, 95% confidence intervals, n.
**Table S4** Comparison between WT and mutant's morphometric parameters.
**Table S5** Inflorescence phenotypes of *tweaky spike* (*tw*) barley mutants and their respective Wild‐Type backgrounds.
**Table S6** Flower phenotypes of *tweaky spike* (*tw*) barley mutants and their respective Wild‐Type backgrounds.
**Table S7** Comparison between WT and mutant's inflorescence/floret phenotype frequencies.
**Table S8** Spike phenotypes of *tw.c1* barley T0 mutant and T0 plant regenerated after transformation, carrying the same T‐DNA as *tw.c1* line, but has no change in *HvAP2L5* gene.
**Table S9** Flower phenotypes of *tw.c1* barley mutant and their WT ‘Golden promise’.
Please note: Wiley is not responsible for the content or functionality of any Supporting Information supplied by the authors. Any queries (other than missing material) should be directed to the *New Phytologist* Central Office.

## Data Availability

Processed data are contained within the article or Supporting Information (Tables S3, S5, S6, S8, S9). Whole‐genome sequencing data are available at NCBI Sequence Read Archive under accession no. PRJNA1446244 (SRR37884601, SRR37884603, SRR37884604, SRR37884605). All code used for SNP and structural variant analyses in this study is publicly available on GitHub at https://github.com/Vejune/Barley_tw.
