# Supplementary material for: Auxin‐related regulation of barley floral organ fate: genetic and phenotypic insights from tweaky spike mutants
Source: New Phytol. 2026 Sep 9;252(3):1166–80. doi: 10.1111/nph.71533 (PMC13628525; doi:10.1111/nph.71533)
Supplement: Supplementary file 1 [file NPH-252-1166-s001.pdf]

New Phytologist Supporting Information

Article title: Auxin-related regulation of barley floral organ fate: genetic and phenotypic insights from *tweaky spike* mutants

Authors: Vėjūnė Pukenytė, Bernadeta Kaminskaitė-Mačė, Miglė Remeikytė, Jurga Jankauskienė, Julius Juodakis, Sigita Jurkonienė, Algė Leistrumaitė, Ljudmilla Timofejeva, Virginija Vaitkūnienė, Justas Stonkus, Raimondas Šiukšta

Article acceptance date: 4 August 2026

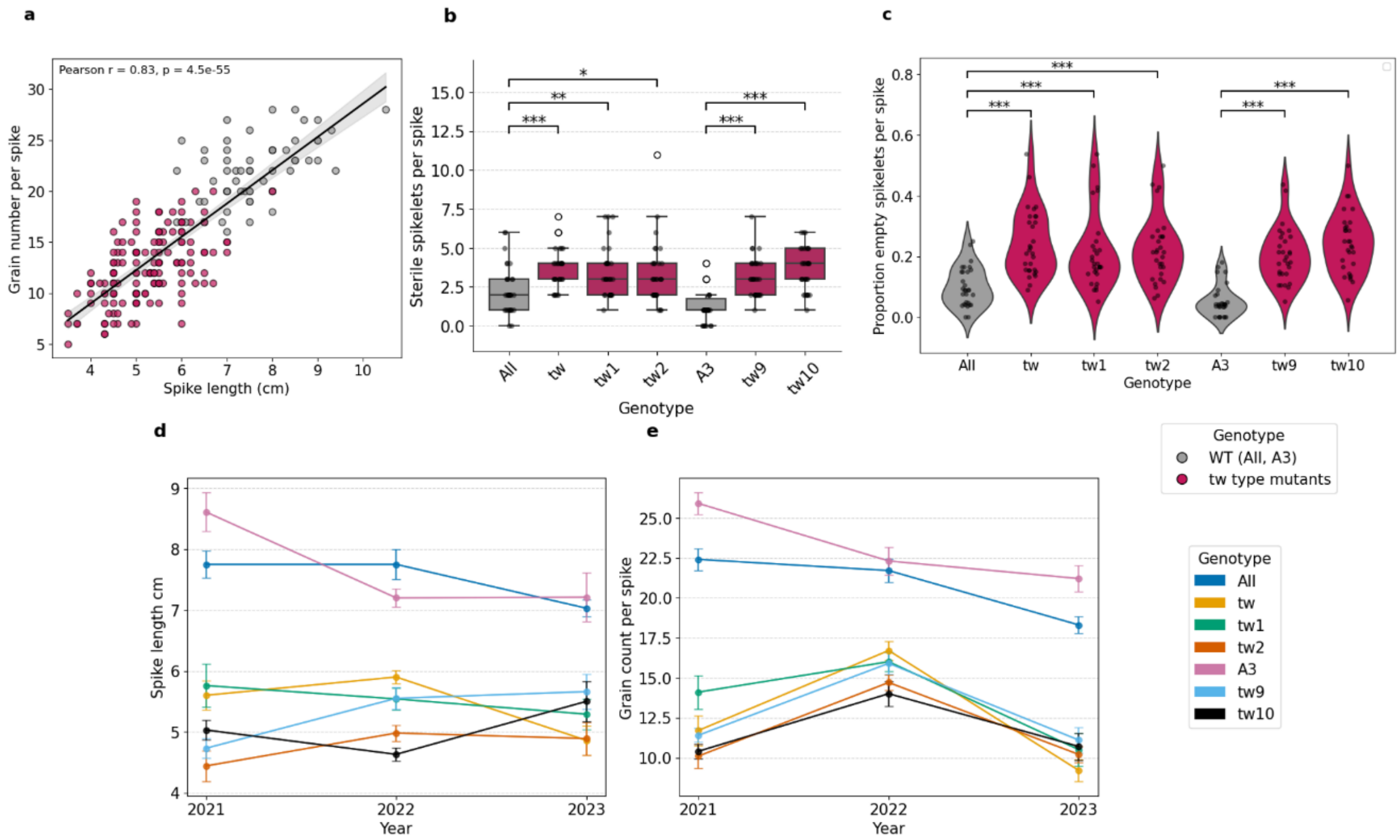

Supplementary Figure 1. Agronomic traits of *tweaky spike* (*tw*) barley (*Hordeum vulgare* L.) mutants and their respective Wild-Type backgrounds, evaluated over three years (2021–2023). (**a**) Pearson correlation between grain count per spike and spike length, (**b**) Sterile spikelet count per spike, data are presented as boxplots (grey colour – WT (AII and A3), pink colour – *tw*-type mutants) showing median, interquartile range, and individual data points represented as translucent black circles, outliers as white circles (n = 30), (**c**) Proportion of empty spikelets per spike, (**d**) Spike length over three years, (**e**) grain count per spike over three years. (n = 30). Wild-Type controls: AII for *tw*, *tw1*, and *tw2*; A3 for *tw9* and *tw10*. Statistical significance was assessed using the Mann–Whitney U test with Benjamini–Hochberg correction for multiple comparisons. Asterisks indicate significant differences compared to the corresponding Wild-Type: *p < 0.05, **p < 0.01, ***p < 0.001.

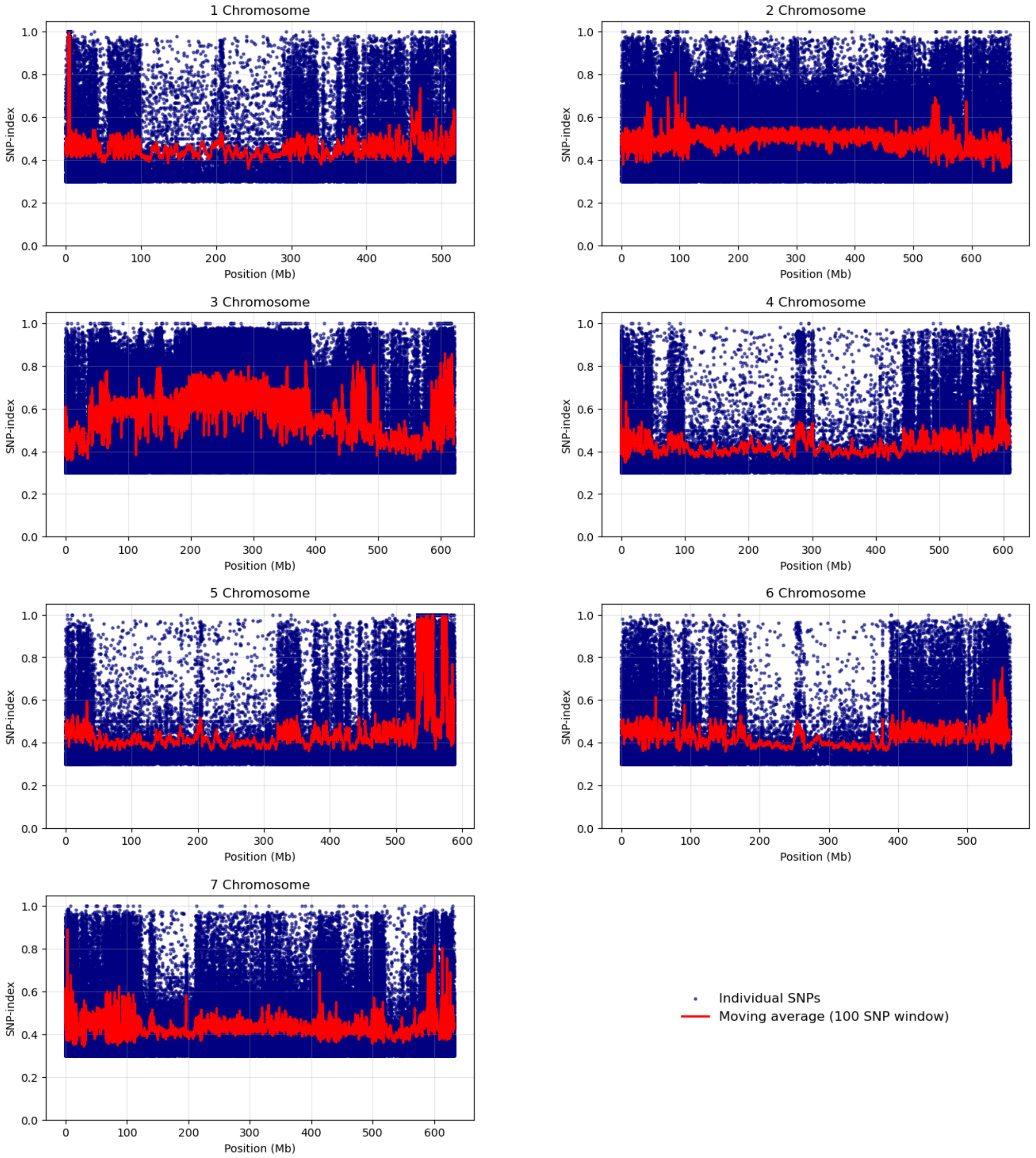

Supplementary Figure 2. Genome-wide distribution of the SNP-index in the *tw-F2* mutant bulk. The SNP-index was calculated as the proportion of reads carrying the mutant allele at each SNP position. Blue dots represent SNP-index values of individual SNPs plotted according to their physical positions across the seven barley chromosomes. The red line represents the moving average of the SNP-index calculated over consecutive windows of 100 SNPs. Chromosome positions are shown in megabases (Mb). Genomic regions in which the SNP-index approaches 1 indicate an increased frequency of alleles derived from the *tw* mutant parent and may therefore represent candidate regions linked to the mutant phenotype.

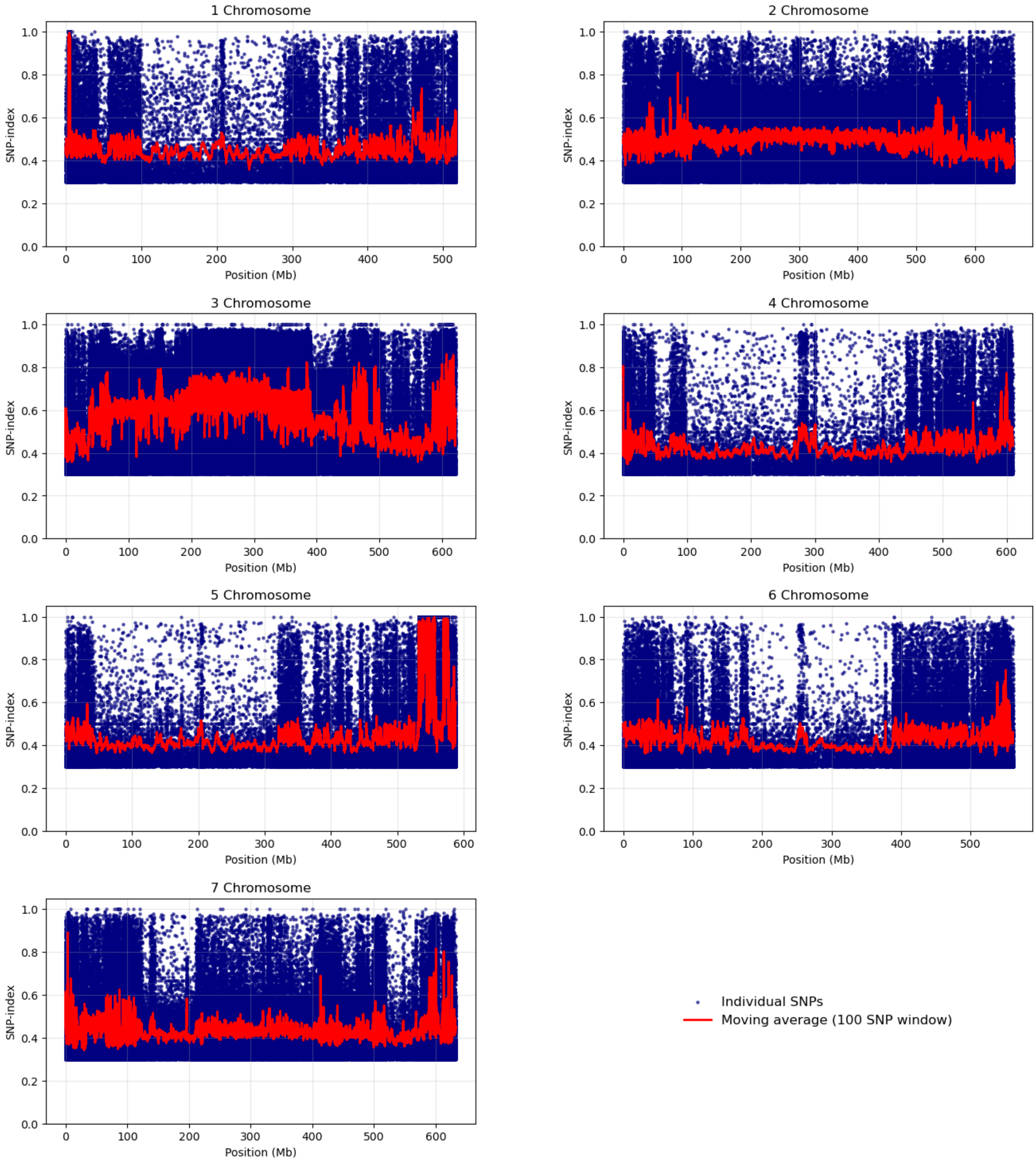

Supplementary Figure 3. Genome-wide distribution of the SNP-index in the *tw2-F2* mutant bulk. The SNP-index was calculated as the proportion of reads carrying the mutant allele at each SNP position. Blue dots represent SNP-index values of individual SNPs plotted according to their physical positions across the seven barley chromosomes. The red line represents the moving average of the SNP-index calculated over consecutive windows of 100 SNPs. Chromosome positions are shown in megabases (Mb). Genomic regions in which the SNP-index approaches 1 indicate an increased frequency of alleles derived from the *tw* mutant parent and may therefore represent candidate regions linked to the mutant phenotype.

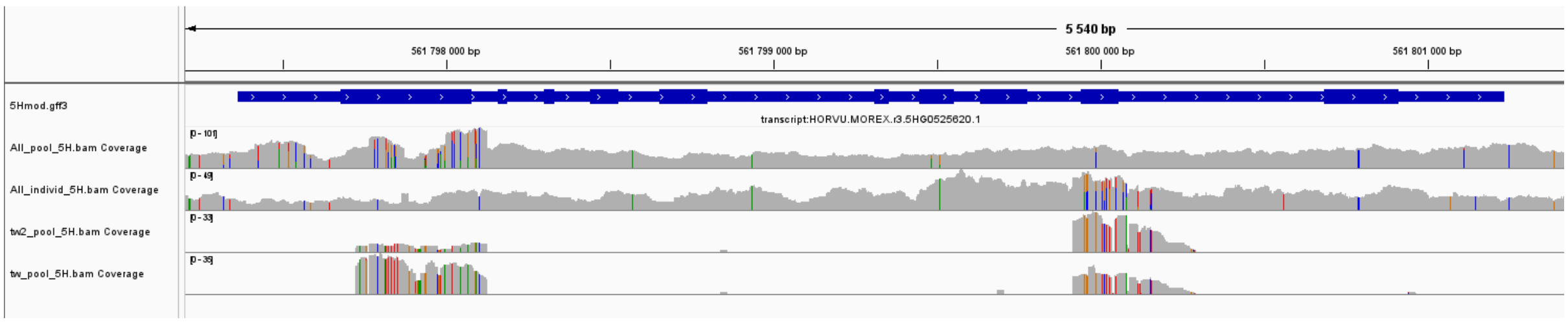

Supplementary Figure 4. Sequencing coverage across the *HvAP2L5* locus in the Wild-Type (WT) and mutant samples. Sequencing reads were aligned to the barley reference genome and visualized using the Integrative Genomics Viewer (IGV). The upper track shows the exon–intron structure of *HvAP2L5*, while the grey coverage profiles are shown for the WT AII pool, the AII individual sample, and the *tw-F2* and *tw2-F2* mutant pools. Continuous read coverage was observed across the *HvAP2L5* locus in the WT samples. In contrast, most of the locus lacked read coverage in *tw-F2* and *tw2-F2* mutant pools, with reads retained only over limited exonic regions, supporting the presence of deletions affecting *HvAP2L5*. Only the *HvAP2L5* locus is shown because the full predicted deletion interval was too large to display coverage.

Supplementary Figure 5. Annotated genomic sequence of HvAP2L5 (*HORVU.MOREX.r3.5HG0525620*) from Morex. Upstream and downstream regions are indicated in blue font. Untranslated regions (5' UTR and 3'UTR) are indicated in green font. Exons are marked in red font. Introns are marked in black font. Primer binding sites indicated in highlighted text.

GGTCAAATCAAGCAACGCAACGGAACCATGTGCTCTAGTGCTTAACAACCGAGGTCACCA 60

CCAAAACCAAAGGAAAAATTATAACGGGGCAACGTCTGGCCGGAAAAGCCAGTCACGTG 120

CGGCGCGCTCCACCTTCCCTCATCGCCGCCTGCAGGTCGCCGCAAGCCGCGCACAAAGGC 180

CGGGTCGACCGGTTCGGCACGCGGTGCCCCGCTCCCCCCGTCCCGATCACCGGGTCGC 240

CTCACCTCACCTCACGGGCTTCCCACCCGGTCAACGTGCTGTACGACACCACCGGCGCAG 300

CCCATTTACGGCGCTGTCGCCGTCGTCCGTCCGTCTCCGTTCCCTCGGGTCCTCTCCTCC 360

CGTGGCCGCGCCGAGAGCCCCACGCGGTGCCGGTGCCGTGGACCGCGGCCGTACGCGAGC 420

CCGTGACGAGGCGAGCGATCCATCGAGGATACATACATAGGTAGCGTAGCGTGGTGCGGA 480

TGGCGTAGCCCGGCGTTGTTTCGGGCGGTGTGGGTTGCATTTGTGGAAACGAATAACGTT 540

GCCGATGGACGGAGCGGTGGACCGGAACGGCACAGTGCCCCGGACAGGTACGCCTGTGGC 600

TGCTGCCGCTGCGTACCCGCCCGCGTGTGGGTCTCCGTCGAATAATTGCACCCCTCCAAT 660

TACACCCGGGTCCCGCCCCGACGAGAAAAAAAACAGTACCCGGACCTAACCTGCAACCTC 720

CAACCCCAAGTAGGAGTAGGAGTACCACCACCGATGGCAGCACAGTACATATATACACGA 780

CGCATACACGAAAATAGAGAGGTGCACGTCGTACTCGTACATACCGGCAGGCAGGCAGAC 840

GCTACCCTGACGCTGCGCTAGACTAGATTGGCCCAGCTGCCGGCTCCCGTCTCGCCAACC 900

GGCCCGGCACGTACCTCACCTGCGCCTTTGGCCCTCCCCTTGGTGTCTGTGTAGGTGTAG 960

CAGCAGCCCCGGCCGTGGTCAAGGGGGAGAAGACGCTACTACACACGAGGCCTCCCAGAT 1020

CGAGCGCGGCATGCATGTATGCATGTGCCGCCGGAGCAAGCGCCGGTACGTATGTACGTA 1080

TATATAATAGCGGCGCCGATTAATCTAGGGTTTGATTAGACTGAGGGGGGCGTGCAGTGC 1140

ACTTGGCTAGTGTAATGCGGTCCTGCGAGGAGGGATCCAATCTTAATCTTAGCTCGTACG 1200

GCCGATGGCTTATCTTAAGCTCGCTAGCTAGGTGGCTAGATAACAACAACAACATGAGTT 1260

GATGGTGCCTCGCGTTAACAATTGCAACGAAAAGCCAACTGCCTGGTGCACACACCGTGG 1320

ACGGATGCGTGGTGGATGGACACCATCATATACCATGGAGCATGGGTGGTTTTTTTTTTC 1380

CTGCTTCCTCTTAAATTAGTTATATGAGCAAAGGGCTTTTGAGTCTTGGTCCATACATGT 1440

CGGTCAAATCAAATCTTAAGACAAAAAGTTGCAGCATCATTTGTTCTAGAATGAAGTTGA 1500

AGTAAATTATGTGCTGCTCTTACGAGTGTTTATGTATTACTACTTTTTTCTTTAGGATGT 1560

ACTGCGAGATTTTTTTAATACGTTGTTGCTTAATGTTTGGTTAGATCGATGACACGGTGT 1620

CATAAAATAATGCCATACCATCGGAGATCTGTTGCAAAGGCACATTGACATATGTAATTG 1680

CACTCTTCGTTGCATTAAAAATATGTTTATGCAGTAATCACGTATTTTTTATAGTTGCAT 1740

CTTTAAAAACATCCAGAGTCTTCGACGTAAGACCTTTCTAGCTGCAGGCCTGATCCGCAT 1800

GAGATGCTGCTTGGTTGTTGTATATGAAATACAATATTATAATGCCCTGTCCTTCATAAA 1860

TTAAATTACTTACATTAGCAAGTTGACATTTATTTATCTTTAGCTTTGAAAACATCCAAC 1920

GCCTTTGGTGTAAGACCTTTCTAGTCGTGCATCAAATTCAGTCAAGATACTACTTGACTG 1980

TTTATAAAATGATAGAATTATAATACATTTTCTACATCAATTAATTATTCAGCATGTCGA 2040

TATTTTATATATTACTTAGCGTTCGCATCCGATACAATACAAAACTGTATGTCGACATAA 2100

AAATAAGTTTCAGTCATTTAACAAGAGGTATATAACACATGATTTGGTACCAAATTCGAG 2160

CTCTTGGTCGATCAAACTAGCGGTCTATGGTGGTGTTTTTTTACCCATGGACTAGACTAA 2220

AAAGTCCCTTGTAGTCCCTAGGTAAAGAAACAGGAGGGACTTTTCCTTAAAGAACTAGGA 2280

AAAGACTTTATTGAAGGGACTTTTTTCTTTAGTCCCTGGGACAAAAAAAAATCTAGTCCC 2340

TTGAAAAGAAACATCACCTATATAAGATCATTTACCTATATTTTTCAGAAAAAGGGATTG 2400

TGGAGAGGAAGCCGGCCAAAAAACCCACGGGGAACCTCTGTTTGATGTTTGATCCCCTCC 2460

GAGAGCGACACGTGGCGTCCGCACCCGGGGCGTGGGTGGCCGTAGCGATTAGCTAGCAGC 2520

GAAGCTCTGCTGCAGGAAAATACGTAGTTTACCTCCCTCTGATGATGCGCTTGGGGGGGC 2580

TTGGCCACCTCGACGTCCCATCTCGCTTTCTTTCTCTTTCTCTTTCCCTTTCTCCCACTG 2640

GCGCAGGGCCCTCTCCTCGTCCTCCTCCAGTCCTCATCCCCATGGCCACCACCACCGCCT 2700

CCACCCCCCACCACTACTTCTACTCCTCCCGCCTCGCCCTCGCAGCCCGCGGCCACCGCG 2760

CTCCCATGCCATAGACGCCAGCCAGCTGATCGGTCGACCTCGCTCGGACGCGGCACGCGG 2820

CGAGCGAGGCGGGCATACCTGCGTTTCATTTGCGAGCTCCCGGCGCGCGGGCCTCGGATC 2880

GGAGGACGGAGATGGTGCTGGATCTCAATGTCGAGTCTCCGGCCGACTCCGGCACGTCCA 2940

GCTCGTCCGTGCTCAACTCCGCGGACGCCGCCGGCGCCTTCCGGTTCGGCCTGCTCGGGA 3000

GCCTCGATGACGACGACTGCTCCGGCGAGCTGGCGCCGGCCGCCGCGTCCGGGTTCGTGA 3060

CGAGGCAGCTGTTCCCCGCGCCGCCGCCCGCGCCCGGGGTCATGATGGGGCAGGCGCCGG 3120

CGCCTCCGCCCACGGCGCCGGTGTGGCAACCTCGGCGCGCCGAGGAGCTGGTCGTGGCGC 3180

AGCGGGTGGCCCCCAAGAAGAAGACGCGGCGGGGGCCGAGGTCGCGCAGCTCGCAGTACC 3240

GGGGCGTCACCTTCTACCGCAGGACCGGCCGCTGGGAGTCGCACATCTGGTTAGCCATCT 3300

CTTCTCGCCATGCCTCTCTACTTACCAAAAGAGTGAACCATTCATTAACTAATCAATCGC 3360

TGATTACACCAGGGATTGCGGGAAGCAGGTCTACTTGGGTGAGCTCAATCAAATCCCAGC 3420

TCCAGCTCCCATCTTCCCCGTGTTTAATTTCGATTGTCTTTGCTATTATTATCATCTTCT 3480

GCTAATTAAAGAAGTTGGTTGATTTCTTCAGGTGGTTTCGACACCGCGCACGCGGCCGCA 3540

AGGTGAATAAATCATCATAAATTAATCAGGCTACGCTTGAACTCCTTTTACCCCGATTTG 3600

AGATTTGACCATGAACTGTGTACTGAGATGAGACGGTGTGGCGTGGCGCAGGGCCTACGA 3660

TCGCGCGGCGATCAAGTTCCGGGGGCTGGAGGCCGACATCAACTTCAATCTGAGCGACTA 3720

CGAGGAGGATTTGAAGCAGGTAATCTTATGAAAGCCAAGCTAGATTGATTGCTGTGCCAC 3780

CCAGTAGTGCGCCGGATGATGATAATATATGGGTGGTGTTGTCCGATGGTTGATATCCGG 3840

TGGGTGGGTGGTGTTTTTTGCCAGATGAGGAACTGGACCAAGGAGGAGTTCGTGCATATC 3900

CTCCGCCGCCAGAGCACCGGGTTCGCCAGGGGGAGCTCGAAGTACCGCGGCGTCACGCTC 3960

CACAAGTGCGGCCGCTGGGAGGCAAGGATGGGCCAACTGCTCGGCAAGAAGTAAGCAGAC 4020

TACACACACGCTAAAATTATATTAACTTCATTCACATTATCATAATTTCCTTAATCAATG 4080

CCATATACTGCAAGATTAGGCTGAAATGAAATTTCATAAACTGTTCATGAACTTGACACT 4140

CATTAGTAGGGTATAGATGTGACAGGGCAGCTGTTCATGCTGTGGACGTTTAATTATCAT 4200

GTCGTAGGTAATCAATCTTAGATTATCTGTTTTGATTGAAACATAATCTTAGCTGGTTTA 4260

GGGTAGGGTCATCAAGCTAATCCATGTTGTTAGCTGTTGGCGCGTTCCTGTGCTGGTGCT 4320

GGTGAGACGTCCACTTCCCCCAACACTCTTATCGCAGACATCTATTTGGAGCAACTGTTA 4380

TAGGTTCCACATATATGATGGAGTTGGCAAAACATGATTAATGGAATCCCTGCACACTAT 4440

AGTTCAGACCCAGAATTGTCAAAGTGATCAATGTGTAGAAGTGATCTCACAGCTGATATA 4500

TCTACATTACCATGACACACAGGTACATATATCTTGGGCTGTTCGACAGCGAAGTTGAAG 4560

CTGCAAGGTGCTTTGATTCGAGCTGATCACTAACATGCGGGTTCACAAAACTTCCTTAAT 4620

TTTCGCTCACTCACTCTCTTGTCATGTTGGGATTTGGTAGAGCGTACGACAGGGCGGCGA 4680

TTCGCTTCAATGGGAGGGATGCTGTGACTAACTTTGATAGTAGCTCCTACAATGGAGATG 4740

CTACACCTGACGTCGAAAATGAGGGTACTACTACAATCAGTCTCACCCGCTGAAATTTTC 4800

CAATCACACCCAACCTTTATCTCATTGTTATCGTTGTTTTCTCAGCAATTGTTGATGCTG 4860

ATGCTCTTGACTTGGATCTAAGGATGTCGCAACCTACCGCGCACGATCCCAAGAGGGACA 4920

ACATCATCGCCGGCCTTCAGTTAACTTTTGATTCCCCTGAGTCGTCAACCACAATGGTCT 4980

CCTCTCAGGTAAAGAAATTATTATGTTCCGTACTAGCTAATCCAGTGGTTTGATTGCTCC 5040

AAAAGATAATTTCCTGTGGCCCAGTTGGTGTCGGTCGATGAGATATTATTGATCCAGGTC 5100

CTATGGCGTATCCTCCTAAACTTACGATGTTTGTGCCTTTTGATATCGCTGTAGCCAATG 5160

AGCTCATCGTCCCAGTGGCCTGTGCATCAACATGGCACAGCAGTACCACCTCAGCAGCAC 5220

CAGCGTTTGTACCCATCTGCTTGTCATGGCTTCTACCCCAACGTACAGGTATCATCACTG 5280

CGAGAACGACCCCCCTCCCCCCTCCTCCTCCTCTGTTTGGCGCGCTGCTCCACCTGCTTA 5340

CTGAAACTGTTGCTGCTGAATAATGTCTCAGAAGAAACTGTTGATAGCATTTCAGTTTTT 5400

GTCAATTTCATCCCTGAAACTAAGGTTTCCATGTTTCTTTCCTATATCCAATCATCCTAA 5460

AACATCTACAAGTAGCATTTTAGGTGTGGTCATGTGTCTACAGACTGAGCTACTTTGCTT 5520

TCTTGAAACGCAAACTTTGATCATTGGCGTCTCTCGATCTCATGGACAGAGAGCATGCTG 5580

TGTCGTCGTACCAGAGTACTTACTAGATGTGGGTGCTACTGTTTGCCCTGTGAGCTAGTA 5640

GATAGATGATTGATGGTTCAATCTGGGTGGATCCGTGAGCGAGCTTGCACCAGCTTTTGA 5700

AAAAACTTGCAGTGCGTCGTCTAGTTCTTACAGTTCCATCCATCCATGACACAGCTTTAG 5760

ATGCAGCTGCTGCTGAGTAGGAGTAGTACCCTGATCACATGGCCCAGCTTTATTCTTGGT 5820

ATATACTGTGCATTCACATGAAAGCAATGCTTTGCTGGATGCACGGCCATGACTTGACAC 5880

TCTCTCTCGGGTTGCAGGTGCAGGTGCAGGAGAGGCCCTTGGAGCCAAGGCCCCCTGAGC 5940

CGTCCTCCTTCCCCGGCTGGGGGTGGCACGCGCAGGCCGTGCCGCCGGGCTCCTCCCACT 6000

CGTTGTTGCTTTATGCTGCAGCATCATCAGGATTCTCTACCGCCGCCGGCGCGAACCCCG 6060

CCCCGCCGCCGTCGTACCCCGACCATCACCACCGCTTCTACTTCCCCCGCCCGCCGGACA 6120

ACTAGCTCTCTGCTTACTCCCATGGACGGTGGTCGACGGGTGTGGGCGTGTGCGCGGGCG 6180

CGATCGACGTGTTCGCGCCTTGTCGGTAACAGTTGTTGTGAATTAATCGGAGAGAGATAA 6240

CATTGCCGAGCCATGTGTCACTGGCTGGTTCCTCTCCCTCGCCATGATCAGAATCACAGG 6300

CATCATGGTTTCCCGTTCAGACTATGTATCCATGCTTTCAATCATGATCATGATCATGCG 6360

GTTGTCCATTACTAGATTCTCATGTATCCATGTTCAAGTTTGTGAAACAGCTGAAAAACC 6420

TTGGAAATTAATGGCGGCAGGTTCATGCGAATGCCATGAATCAAACAGCAACGCGCCCTT 6480

GCGTTAACCGTTGGGGCTTTCTGACTGCGGTGCCATGACTACACCACCATCACCATTGTT 6540

GCGTTAACCGTTGGGCTTTTTCTGAACGACGTGCGTCGATTGACAGGTGGGTCCCCGGAC 6600

CCGACGGCTGTGGCCAGGCAGCCAGTGAGGCAGCGAGTAGGCGAGTAGAGTACTAGGCGG 6660

TCAGTGATTGTTCGTTGGCATCCTCCGTTCTGGGAGCAACCTCTGATTGATGGGCCGTAA 6720

CCGCAGCCATCACCTCACTCTGAGCCAAACCTCCTCCCTCTTCTCTCTCTCTCTCTCTCT 6780

CCCCCCCCCCCCCCCCGAAGAAATCCATTCAATCCGGCACAGTGAAGTGAAAGGCTTTGC 6840

CACCCACCCAACACATCCACAATGGCAAAGCTACTTGTACCAGCAGGCAGCAGCAGCAGT 6900

ACTGTGGCAGTAGTAAGCTGTAGGCTGTAGCTGCGCGTGGGCTCGCTCTCTGGCTGGCTG 6960

GCTGCGAGCACTGGAGCGGCACAGTGCGGCCCGCCCGGAGCGGCTTCTCGTCCGCGGCCC 7020

CCAATGGCGCCGCGGCGCGTGTGCAGTGC 7049

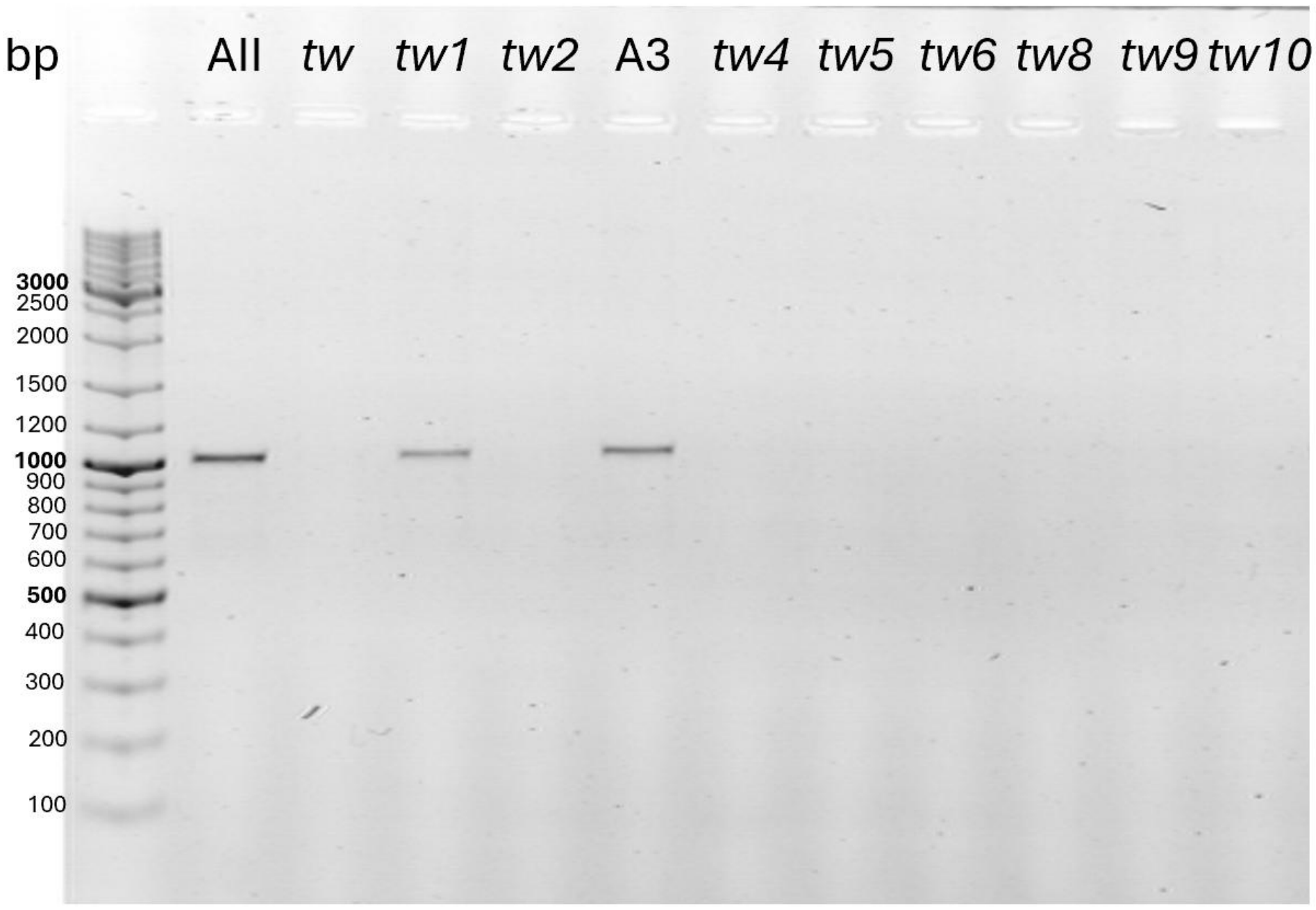

Supplementary Figure 6. Amplification of fragment W4 from genomic DNA using primer pair AP2L5/W4. A single band of 1018 bp was observed in AII, tw1, and A3 samples. No band was observed in tw, tw2, or tw4-10 samples. AII and A3 are wild-type genotype samples.

Supplementary Figure 7. 'Auksiniai II' (AII) and *tw1 HvAP2L5* coding sequence alignment (HORVU.MOREX.r3.5HG0525620) location: 5H: 561,797,365-561,801,237.

```
AII     ATGGTGCTGGATCTCAATGTCGAGTCTCCGGCCGACTCCGGCACGTCCAGCTCGTCCGTG   60
tw1     ATGGTGCTGGATCTCAATGTCGAGTCTCCGGCCGACTCCGGCACGTCCAGCTCGTCCGTG   60

AII     CTCAACTCCGCGGACGCCGCCGGCGCCTTCCGGTTCGGCCTGCTCGGGAGCCCCGATGAC  120
tw1     CTCAACTCCGCGGACGCCGCCGGCGCCTTCCGGTTCGGCCTGCTCGGGAGCCCCGATGAC  120

AII     GACGACTGCTCCGGCGAGCTGGCGCCGGCCGCCGCGTCCGGGTTCGTGACGAGGCAGCTG  180
tw1     GACGACTGCTCCGGCGAGCTGGCGCCGGCCGCCGCGTCCGGGTTCGTGACGAGGCAGCTG  180

AII     TTCCCCGCGCCGCCGCCCGCGCCCGGGGTCATGATGGGGCAGGCGCCGGCGCCTCCGCCC  240
tw1     TTCCCCGCGCCGCCGCCCGCGCCCGGGGTCATGATGGGGCAGGCGCCGGCGCCTCCGCCC  240

AII     ACGGCGCCGGTGTGGCAACCTCGGCGCGCCGAGGAGCTGGTCGTGGCGCAGCGGGTGGCC  300
tw1     ACGGCGCCGGTGTGGCAACCTCGGCGCGCCGAGGAGCTGGTCGTGGCGCAGCGGGTGGCC  300

AII     CCCAAGAAGAAGACGCGGCGGGGGCCGAGGTCGCGCAGCTCGCAGTACCGGGGCGTCACC  360
tw1     CCCAAGAAGAAGACGCGGCGGGGGCCGAGGTCGCGCAGCTCGCAGTACCGGGGCGTCACC  360

AII     TTCTACCGCAGGACCGGCCGCTGGGAGTCGCACATCTGGGATTGCGGGAAGCAGGTCTAC  420
tw1     TTCTACCGCAGGACCGGCCGCTGGGAGTCGCACATCTGGGATTGCGGGAAGCAGGTCTAC  420

AII     TTGGGTGGTTTCGACACCGCGCACGCGGCCGCAAGGGCCTACGATCGCGCGGCGATCAAG  480
tw1     TTGGGTGGTTTCGACACCGCGCACGCGGCCGCAAGGGCCTACGATCGCGCGGCGATCAAG  480

AII     TTCCGGGGGCTGGAGGCCGACATCAACTTCAATCTGAGCGACTACGAGGAGGATTTGAAG  540
tw1     TTCCGGGGGCTGGAGGCCGACATCAACTTCAATCTGAGCGACTACGAGGAGGATTTGAAG  540

AII     CAGATGAGGAACTGGACCAAGGAGGAGTTCGTGCATATCCTCCGCCGCCAGAGCACCGGG  600
tw1     CAGATGAGGAACTGGACCAAGGAGGAGTTCGTGCATATCCTCCGCCGCCAGAGCACCGGG  600

AII     TTCGCCAGGGGGAGCTCGAAGTACCGCGGCGTCACGCTCCACAAGTGCGGCCGCTGGGAG  660
tw1     TTCGCCAGGGGGAGCTCGAAGTACCGCGGCGTCACGCTCCACAAGTGCGGCCGCTGGGAG  660


AII     GCAAGGATGGGCCAACTGCTCGGCAAGAAGTACATATATCTTGGGCTGTTCGACAGCGAA  720
tw1     GCAAGGATGGGCCAACTGCTCGGCAAGAAGTACATATATCTTGGGCTGTTCGACAGCGAA  720

AII     GTTGAAGCTGCAAGAGCGTACGACAGGGCGGCGATTCGCTTCAATGGGAGGGATGCTGTG  780
tw1     GTTGAAGCTGCAAGAGCGTACGACAGGGCGGCGATTCGCTTCAATGGGAGGGATGCTGTG  780

AII     ACTAACTTTGATAGTAACTCCTACAATGGAGATGCTACACCTGACGTCGAAAATGAGGCA  840
tw1     ACTAACTTTGATAGTAACTCCTACAATGGAGATGCTACACCTGACGTCGAAAATGAGGCA  840

AII     ATTGTTGATGCTGATGCTCTTGACTTGGATCTAAGGATGTCGCAACCTACCGCGCACGAT  900
tw1     ATTGTTGATGCTGATGCTCTTGACTTGGATCTAAGGATGTCGCAACCTACCGCGCACGAT  900

AII     CCCAAGAGGGACAACATCATCGCCGGCCTTCAGTTAACTTTTGATTCCCCTGAGTCGTCA  960
tw1     CCCAAGAGGGACAACATCATCGCCGGCCTTCAGTTAACTTTTGATTCCCCTGAGTCGTCA  960

AII     ACCACAATGGTCTCCTCTCAGCCAATGAGCTCATCGTCCCAGTGGCCTGTGCATCAACAT 1020
tw1     ACCACAATGGTCTCCTCTCAGCCAATGAGCTCATCGTCCCAGTGGCCTGTGCATCAACAT 1020

AII     GGCACAGCAGTACCACCTCAGCAGCACCAGCGTTTGTACCCATCTGCTTGTCATGGCTTC 1080
tw1     GGCACAGCAGTACCACCTCAGCAGCACCAGCGTTTGTACCCATCTGCTTGTCATGGCTTC 1080

AII     TACCCCAACGTACAGGTGCAGGTGCAGGAGAGGCCCTTGGAGCCAAGGCCCCCTGAGCCG 1140
tw1     TACCCCAACGTACAGGTGCAGGTGCAGGAGAGGCCCTTGGAGCCAAGGCCCCCTGAGCCG 1140
```

AII	TCCTCCTTCCCCGGCTGGGGGTGGCACGCGCAGGCCGTGCCGCCGGGCTCCTCCCACTCG	1200
tw1	TCCTCCTTCCCCGGCTGGGGGTGGCACGCGCAGGCCGTGCCGCCGGGCTCCTCCCACTCG	1200
AII	CCGTTGCTTTATGCTGCAGCATCATCAGGATTCTCTACCGCCGCCGGCGCGAACCCCGCC	1260
tw1	CCGTTGCTTTATGCTGCAGCATCATCAGGATTCTCTACCGCCGCCGGCGCGAACCCCGCC	1260
AII	CCGCCGCCGTCGTACCCCGACCATCACCACCGCTTCTACTTCCCCCGCCCGCCGGACAAC	1320
tw1	CCGCCGCCGTCGTACCCCGACCATCACCACCGCTTCTACTTCCCCCGCCCGCCGGACAAC	1320
AII	TAG	1323
tw1	TAG	1323

Supplementary Figure 8. Sequence alignment of the *HvAP2L5* gene (*HORVU.MOREX.r3.5HG0525620*) upstream region from 'Auksiniai II', 'Auksiniai 3', and *tw1*. The upstream region is shown in black, the 5′ untranslated region (5′ UTR) is highlighted in light green, and the translation initiation codon (ATG) is underlined.

```
AII      ------------------------------------------------------------
A3       ------------------------------------------------------------
tw1      CGCAACGGAACCATGCGCCCTAGTGCTTAACAACCGAGGTCACCACCAAAATCAAAGGAA    60

AII      -----GAAAGATAACGGGGCAACCTCTGGCCGGAAAAGCCAGTCACGTGCGGCGCGCTCC    55
A3       --------TTATAACGGGGCAACGTCTGGCCGGAAAAGCCAGTCACGTGCGGCGCGCTCC    52
tw1      AAAAAGAAAGATAACGGGGCAACCTCTGGCCGGAAAAGCCAGTCACGTGCGGCGCGCTCC   120

AII      ACCTTCCCTCATCGCCGCCTGCAGGTCGCCGCAAGCCGCGCACAAAGGCCGGGTCGACCG   115
A3       ACCTTCCCTCATCGCCGCCTGCAGGTCGCCGCAAGCCGCGCACAAAGGCCGGGTCGACCG   112
tw1      ACCTTCCCTCATCGCCGCCTGCAGGTCGCCGCAAGCCGCGCACAAAGGCCGGGTCGACCG   180

AII      GTTCGGCACGCGGTGCCCCCGCTCCCCCCCGTCCCGATCACCGGGTCGCCTCACCTCACG   175
A3       GTTCGGCACGCGGTGCCCCCGCTCCCCCCCGTCCCGATCACCGGGTCGCCTCACCTCACG   172
tw1      GTTCGGCACGCGGTGCCCCCGCTCCCCCCCGTCCCGATCACCGGGTCGCCTCACCTCACG   240

AII      GGCTTCCCACCCGGTCAACGTGCTGTACCACACCACCGGCGCAGCCCATTTACGGCGCTG   235
A3       GGCTTCCCACCCGGTCAACGTGCTGTACCACACCACCGGCGCAGCCCATTTACGGCGCTG   232
tw1      GGCTTCCCACCCGGTCAACGTGCTGTACCACACCACCGGCGCAGCCCATTTACGGCGCTG   300

AII      TCGCCGTCGTCCGTCCGTCTCCGTTCCCTCGGGTCCTCTCTTCCCGTGGCCGCGCCGAGA   295
A3       TCGCCGTCGTCCGTCCGTCTCCGTTCCCTCGGGTCCTCTCCTCCCGTGGCCGCGCCGAGA   292
tw1      TCGCCGTCGTCCGTCCGTCTCCGTTCCCTCGGGTCCTCTCTTCCCGTGGCCGCGCCGAGA   360

AII      GCCCCACGCGGTGCCGGTGCCGTGGACCGCGGCCGTACGCGAGCCCGTGACGAGGCGAGC   355
A3       GCCCCACGCGGTGCCGGTGCCGTGGACCGCGGCCGTACGCGAGCCCGTGACGAGGCGAGC   352
tw1      GCCCCACGCGGTGCCGGTGCCGTGGACCGCGGCCGTACGCGAGCCCGTGACGAGGCGAGC   420

AII      GATCCATCGAGGATACATACATAGGTAGCGTAGCGTGGTGCGGATGGCGTAGCCCGGCGT   415
A3       GATCCATCGAGGATACATACATAGGTAGCGTAGCGTGGTGCGGATGGCGTAGCCCGGCGT   41
tw1      GATCCATCGAGGATACATACATAGGTAGCGTAGCGTGGTGCGGATGGCGTAGCCCGGCGT   480

AII      TGTTTCGGGCGGTGTGGGTTGCATTTGTGGAAACGAATAACGTTGCCGATGGACGGAGCG   475
A3       TGTTTCGGGCGGTGTGGGTTGCATTTGCGGAAACGAATAACGTTGCCGATGGACGGAGCG   472
tw1      TGTTTCGGGCGGTGTGGGTTGCATTTGTGGAAACGAATAACGTTGCCGATGGACGGAGCG   54

AII      GTGGACCGGAACGGCACAGTACCCCGGACAGGTACGCCTGTGGCTGCTGCCGCTGCGTAC   535
A3       GTGGACCGGAACGGCACAGTGCCCCGGACAGGTTCGCCTGTGGCTGCTGCCGCTGCGTAC   532
tw1      GTGGACCGGAACGGCACAGTACCCCGGACAGGTACGCCTGTGGCTGCTGCCGCTGCGTAC   600

AII      CCGCCCGCGTGTGGGTCTCCGTCGAATAATTGCACCCGTCCAATTACACCCGGGTCCCGC   595
A3       CCGCCCGCGTGTGGGTCTCCGTCGAATAATTGCACCCGTCCAATTACACCCGGGTCCCGC   59
tw1      CCGCCCGCGTGTGGGTCTCCGTCGAATAATTGCACCCGTCCAATTACACCCGGGTCCCGC   660

AII      CCCGACGAGAAAAAAAAACAGTACCCGGACCTAACCTGCAACCTCCAACCCCAAGTAGGAG   655
A3       CCCGACGAGAAAAAAAAACAGTACCCAGACCTAACCTGCAACCTCCAACCCCAAGTAGGAG   652
tw1      CCCGACGAGAAAAAAAAACAGTACCCGGACCTAACCTGCAACCTCCAACCCCAAGTAGGAG   720

AII      TAGGAGTACCACCACCGATGGCAGCACAGTACATATATACACGACGCATACACGAAAATA   715
A3       TAGGAGTACCACCACCGATGGCAGCACAGTACATATATACACGACGCATACACGAAAATA   712
tw1      TAGGAGTACCACCACCGATGGCAGCACAGTACATATATACACGACGCATACACGAAAATA   780

AII      GAGAGGTGCACGTCGTACTCGTACATACCGGCAGGCAGGCAGACGCTACCCTGACGCTGC   775
A3       GAGAGGTGCACGTCGTACTCGTACATACCGGCAGGCAGGCAGACGCTACCCTGACGCTGC   772
```

```
tw1     GAGAGGTGCACGTCGTACTCGTACATACCGGCAGGCAGGCAGACGCTACCCTGACGCTGC   840


AII     GCTAGACTAGATTGGCCCAGCTGCCGGCTCCCGTCTCGCCAACCGGCCCGGCACGTACCT   835
A3      GCTAGACTAGATTGGTCCGGCTGCCGGCTCCCGTCTCGCCAACCGGCCCGGCACGTACCT   832
tw1     GCTAGACTAGATTGGCCCAGCTGCCGGCTCCCGTCTCGCCAACCGGCCCGGCACGTACCT   900

AII     CACCTGCGCCTTTGGCCCTCCCCTTGGTGTCTGTGTAGGTGTAGCAGCAGCCCCGGCCGT   895
A3      CACCTGCGCCTTTGGCCCTCCCCTTGGTGTCTGTGTAGGTGTAGCGGCAGCCCCGGCCGT   892
tw1     CACCTGCGCCTTTGGCCCTCCCCTTGGTGTCTGTGTAGGTGTAGCAGCAGCCCCGGCCGT   960

AII     GGTCAAGGGGGAGAAGACGCTACTACACACGAGGCCTCCCAGATCGAGCGCGGCATGCAT   955
A3      GGTCAAGGAAGAGAAGACACTACTACACACGAGGCCTCCCAGATCGGGCGCGGCAAGCAT   952
tw1     GGTCAAGGGGGAGAAGACGCTACTACACACGAGGCCTCCCAGATCGAGCGCGGCATGCAT  1020

AII     GTATGCATGTGCCGCCGGAGCAAGCGCCGGTACGTATGTACGTATATATAATAGCGGCGC  1015
A3      GTATGCATGTGCCGCCGGAGCAAGCGCCGGTACGTATGTACGTATATATAATAGCGGCGC  1012
tw1     GTATGCATGTGCCGCCGGAGCAAGCGCCGGTACGTATGTACGTATATATAATAGCGGCGC  108

AII     CGATTAATCTAGGGTTTGATTAGACTGAGGGGGGCGTGCAGTGCACTTGGCTAGTGTAAT  1075
A3      CGATTAATCTAGGGTTTGATTAGACTGAGGGGGGCGTGCAGTGCACTTGGCTAGTGTAAT  1072
tw1     CGATTAATCTAGGGTTTGATTAGACTGAGGGGGGCGTGCAGTGCACTTGGCTAGTGTAAT  1140

AII     GCGGTCCTGCGAGGAGGGATCCAATCTTAATCTTAGCTCGTACGGCCGATGGCTTATCTT  1135
A3      GCGGTCCTGCGAGGAGGGATCCAATTTTAATTTTAGCTTGTACGGCCGATGGCTTATTTT  1132
tw1     GCGGTCCTGCGAGGAGGGATCCAATCTTAATCTTAGCTCGTACGGCCGATGGCTTATCTT  1200

AII     AAGCTCGCTAGCTAGGTGGCTAGATAACAACAACAACATGAGTTGATGGTGCCTCGCGTT  1195
A3      AAGCTCGCTAGCTAGGTGGCTAGATAACAACAACAACATGAGTTGATGGTGCCTCGCGTT  1192
tw1     AAGCTCGCTAGCTAGGTGGCTAGATAACAACAACAACATGAGTTGATGGTGCCTCGCGTT  1260

AII     AACAATTGCAACGAAAAGACAACTGCCCGGTGCACACACCGTGGACGGATGCGTGGTGGA  1255
A3      AACAATTGCAACGAAAAGACAACTGCCCGGTGCACACACCGTGGACGGATGCGTGGTGGA  1252
tw1     AACAATTGCAACGAAAAGACAACTGCCCGGTGCACACACCGTGGACGGATGCGTGGTGGA  1320

AII     TGGACACCATCATATACCATGGAGCATGGGTGG-TTTTTTTTTTCTCTGCTTTCTCTTAA  1314
A3      TGGACACCATCATATACCATGGAGCATGGGTGGTTTTTTTTTTTCTCTGCTTTCTCTTAA  1312
tw1     TGGACACCATCATATACCATGGAGCATGGGTGG-TTTTTTTTTTCTCTGCTTTCTCTTAA  1379

AII     ATTAGTTATATGAGCAAAGGGCTTTTGAGTCTTGGTCCATACATGTCGGTCAAATCAAAT  1374
A3      ATTAGTTATATGAGCAAAGGGCTTTTGAGTCTTGGTACATACATGTCGGTCAAATCAAAT  1372
tw1     ATTAGTTATATGAGCAAAGGGCTTTTGAGTCTTGGTCCATACATGTCGGTCAAATCAAAT  1439

AII     CTTGGGACAGAAAGTTGCAGCATCATTTGTTCTAGAATGAAGTTGAAATAAATTATGTGC  1434
A3      CTTGGGACAGAAAGTTGCAGCATCATTTGTTCTAGAATGAAGTTGAAGTAAATTATGTGC  1432
tw1     CTTGGGACAGAAAGTTGCAGCATCATTTGTTCTAGAATGAAGTTGAAATAAATTATGTGC  1499

AII     TGCACTTACAAGTGTTTATGTATTACTACCTTTCTTCTTAGGATGTACTGCGAGGTTTTT  1494
A3      TGCACTTAC-AGTGTTTATGTATTACTACTTTTCTTCTTAGGATGTACTGCGAGGTTTTT  1491
tw1     TGCACTTACGAGTGTTTATGTATTACTACCTTTCTTCTTAGGATGTACTGCGAGGTTTTT  1559

AII     TTAGTACGTTGTTGCTTGATGTTTGGTTAAATCGATGACACGGTGTCATAAAATAATGCC  1554
A3      TTAGTACGTTGTTGCTTGATGTTTGGTTAAATCGATGACACGGTGTCATAAAATAATGCC  1551
tw1     TTAGTACGTTGTTGCTTGATGTTTGGTTAAATCGATGACACGGGGTCATAAAATAATGCC  1619

AII     -----ATGTCATCGGAGATCTGTTGCAAAGGCACATTGACATATGTACTTGCACTCTTCG  1609
A3      ATACCATGCCATCGGAGATCTGTTGCAAAGGCACATTGACATATGTACTTGCACTCTTCG  1611
tw1     -----ATACCATCGGAGATCTGTTGCAAAGGCACATTGACATATGTACTTGCACTCTTCG  1674


AII     TTGCACTAAAAATATATTTATGTAGTACTCACGTATTTTTTATAGTTGCATCTTTGAAAA  1669
```

```
A3       TTGCACTAAAAATATATTTATGTAGTACTCAAGTATTTTTTATAGTTGCATCTTTGAAAA  1671
tw1      TTGCACTAAAAATATATTTATGTAGTACTCACGTATTTTTTATAGTTGCATCTTTGAAAA  1734

AII      CATCCAGAGTCTTCGACGCAAGACCTTTCTAGCTGCATGCCTGATCCGCATGAGATGCTG  1729
A3       CATCCAGAGTCTTCGACGCAAGACCTTTCTAGCTGCATGCCTGATCCGCATGAGATGCTG  1731
tw1      CATCCAGAGTCTTCGACGCAAGACCTTTCTAGCTGCATGCCTGATCCGCATGAGATGCTG  1794

AII      CTTGGTTGTTGCATATAAAATACAATATTATAATGCTCTGTCCTTCATAAATTAAATTAC  1789
A3       CTTGGTTGTTGCATATAAAATACAATATTATAATGCTCTGTCCTTCATAAATTAAATTAC  1791
tw1      CTTGGTTGTTGCATATAAAATACAATATTATAATGCTCTGTCCTTCATAAATTAAATTAC  1854

AII      TTACATTAGCAAGTTGACATTTATTTATCTTTAGCTTTGAAAACGTCCAACATCTTTGGT  1849
A3       TTACATTAGCAAGTTGACATTTATTTATCTTTAGCTTCGAAAACGTCCAACATCTTTGGT  1851
tw1      TTACATTAGCAAGTTGACATTTATTTATCTTTAGCTTTGAAAACGTCCAACATTTTTGGT  1914

AII      GTACGACCTTTCTAGTCGTGCATCAAATTCAGACAAGATGCTACTTGACCGTTTATAAAA  1909
A3       GTACGACCTTTCTAGTCGTGCATCAAATTCAGACAAGATGCTACTTGACCGTTTATAAAA  1911
tw1      GTACGACCTTTCTAGTCGTGCATCAAATTCAGACAAGATGCTACTTGACCGTTTATAAAA  1974

AII      TGATAGAACTATAATACACTTTTTACATTAATTAATTATTCAACATGTCGATATTTTATA  1969
A3       TGATAGAACTATAATACACTTTTTACATTAATTAATTATTCAACATGTCGATATTTTATA  1971
tw1      TGATAGAACTATAATACACTTTTTACATTAATTAATTATTCAACATGTCGATATTTTATA  2034

AII      TATTACTTAGCATTCGCATCCGATACAATACAAAACTGTATGTTGACATAAAAATAAGTT  2029
A3       TATTACTTAGCATTCGCATCCGATACAATACAAAACTGTATGTTGACATAAAAATAAGTT  2031
tw1      TATTACTTAGCATTCGCATCCGATACAATACAAAACTGTATGTTGACATAAAAATAAGTT  2094

AII      TCAGTCATTTAACAGAGGTACCAAATTCAAGCTCTTGATCGATCAAACTAGCAGCTTATA  2089
A3       TCAGTCATTTAACAGAGGTACCAAATTCAAGCTCTTGATCGATCAAACTAGCAGCTTATA  2091
tw1      TCAGTCATTTAACAGAGGTACCAAATTCAAGCTCTTGATCGATCAAACTAGCAGCTTATA  2154

AII      TAAAATCATTTACCTATGTTTTTTAGAAAAAAAAAAGGGGGGCATTTAGCTTGTGGAGAG  2149
A3       TAAAATCATTTACCTATGTTTTTTTTTTAAAAA-----GGGAGCATTTAGCTTGTGGAGAG  2146
tw1      TAAAATCATTTACCTATGTTTTTTAGAAAAAAAAAAGGGGGGCATTTAGCTTGTGGAGAG  2214

AII      GAAGCCGGCCAAAAAACCCAAGGGAAACCTCTGTTTGATGTTTGATCCCCTCCTAGAGCG  2209
A3       GAAGCCGGCCAAAAAACCCAAGGGAAACCTCTGTTTGATGTTTGATCCCCTCCTAGAGCG  2206
tw1      GAAGCCGGCCAAAAAACCCAAGGGAAACCTCTGTTTGATGTTTGATCCCCTCCTAG----  2270

AII      ACACGTGGCGTCCGCACCCGGGGCGTGGGTGGCCGTAGCGATTAGCTAGCAGCGAAGCTC  2269
A3       ACACGTGGCGTCCGCACCCAGGGCGTGGGTGGCCGTAGCGATTAGCTAGCAGCGAAGC--  2264
tw1      ------------------------------------------------------------  2270


AII      TGCTGCACGAAAGCTGCACGAAAACAAAAAATACGTAGTTTACTTCCCTCTGATGATGCG  2329
A3       ------------------------------------------------------------  2264
tw1      ------------------------------------------------------------  2270


AII      CTTGGGGTTGGGCTTGGCCACCTCGACGTCCCATCTCGCTTTCTTTCTCTTTCTCTTTCC  2389
A3       ----------GGCTTGGCCACCTCGACGTCCCATCTCGCTTTCTTTCTCTTTCTCTTTCC  2314
tw1      ----------GGCTTGGCCACCTCGACGTCCCATCTCGCTTTCTTTCTCTTTCTCTTTCC  2320

AII      CTTTCTCCCACTGGCGCAGGGCCCTCTCCTCGTCCTCCTCCAGTCCTCATCCCCATGGCC  2448
A3       CTTTCTCCCACTGGCGCAGGGCCCTCTCCTCGTCCTCCTCCAGTCCTCATCCCCATGGCC  2375
tw1      CTTTCTCCCACTGGCGCAGGGCCCTCTCCTCGTCCTCCTCCAGTCCTCATCCCCATGGCC  2380

AII      ACCACCACCGCCTCCACCCCCCACCACTACTTCTACTCCTCCCGCCTCGCCCTCGCAGCC  2509
A3       ACCACCACCGCCTCCACCCCCCACCACTACTTCTACTCCTCCCGCCTCGCCCTCGCAGCC  2434
tw1      ACCACCACCGCCTCCACCCCCCACCACTACTTCTACTCCTCCCGCCTCGCCCTCGCAGCC  2440
```

```
AII        CGCGGCCACCGCGCTCCCATGCCATAGACGCCACCCAGCTGATCGGTCGAGCTCGCTCGG  2569
A3         CGCGGCCACCGCGCTCCCATGCCATAGACGCCAGCCAGCTGATCGGTCGACCTCGCTCGG  2494
tw1        CGCGGCCACCGCGCTCCCATGCCATAGACGCCACCCAGCTGATCGGTCGAGCTCGCTCGG  2500

AII        -------ACGCGGCACGCGGCGAGCGAGGCGGGCATACCTGCGTTTCATTTGCGAGTTCC  2622
A3         ACGCGGCACGCGGCACGCGGCGAGCGAGGCGGGCATACCTGCGTTTCATTTGCGAGTTCC  2554
tw1        -------ACGCGGCACGCGGCGAGCGAGGCGGGCATACCTGCGTTTCATTTGCGAGTTCC  2553

AII        CGGCGCGCGGGCCTCGGATCGGAGGACGGAGATG                            2656
A3         CGGCGCGCGGGCCTCGGATCGGAGGACGGAGATG                            2588
tw1        CGGCGCGCGGGCCTCGGATCGGAGGACGGAGATG                            2587
```

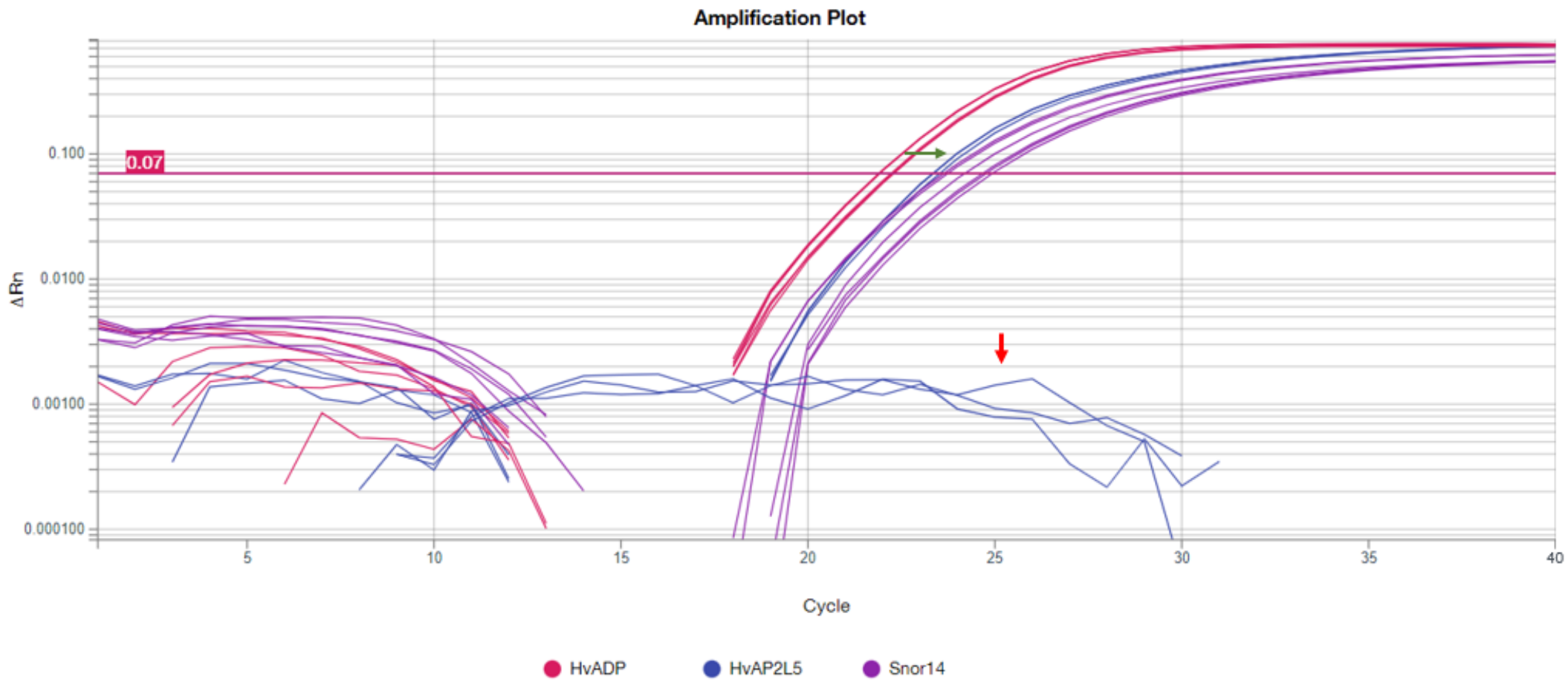

Supplementary Figure 9. Log-scaled RT-qPCR amplification curves of the target gene *HvAP2L5* (*HORVU.MOREX.r3.5HG0525620*) and the reference genes *HvADP (HORVU.MOREX.r3.6HG0606370)* and *Snor14 (LOC123445868)* in coleoptile tissue of Wild-Type 'Auksiniai II' and *tw1* mutant plants. Wild-Type plants show robust amplification of *HvAP2L5* (green arrow), whereas the *tw1* mutant shows no detectable transcript accumulation (red arrow). Reference genes amplified consistently in both genotypes, confirming RNA quality and successful cDNA synthesis. The horizontal line represents the 0.07 ΔRn threshold used to determine the onset of exponential amplification.

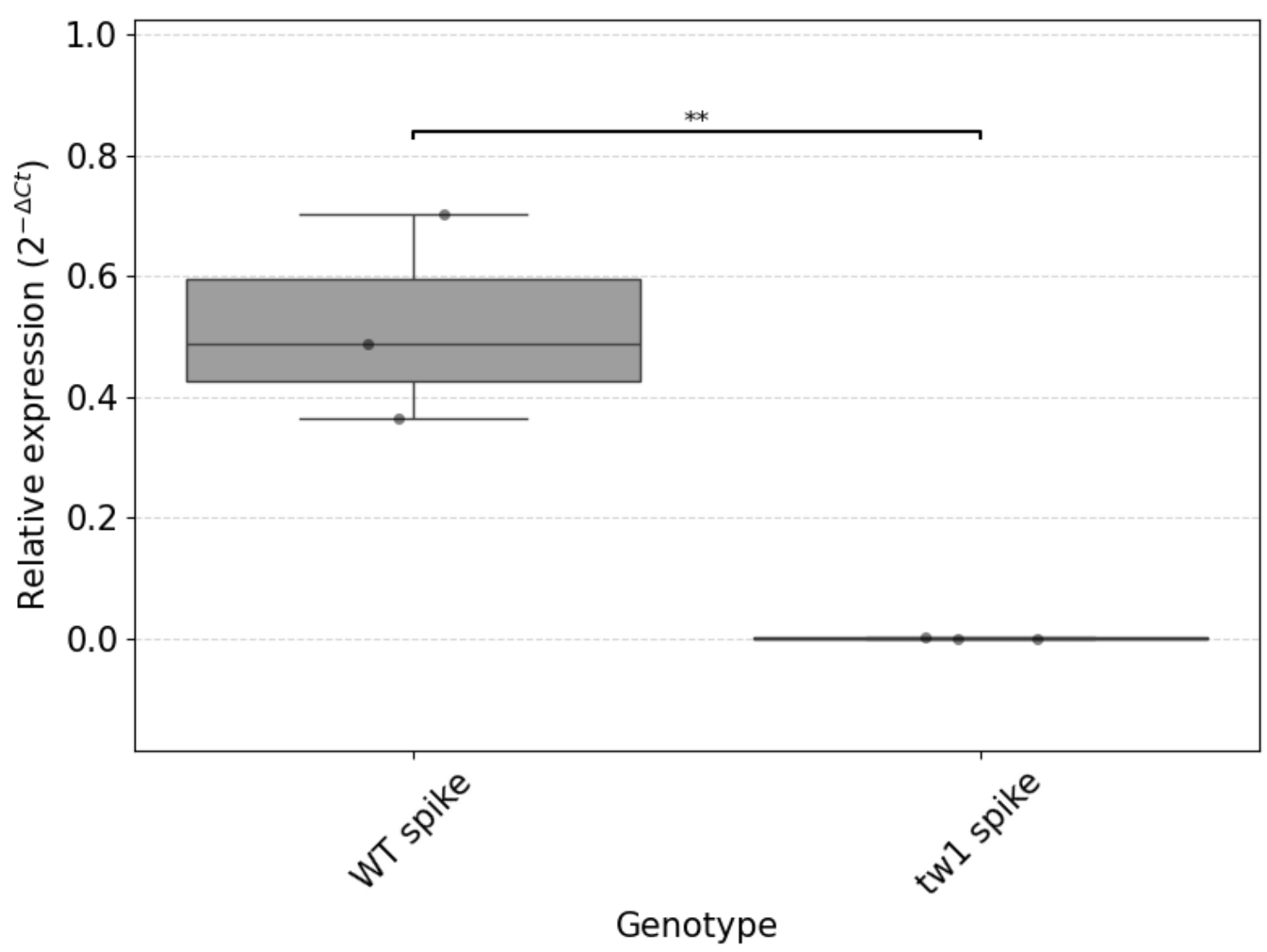

Supplementary Figure 10. Relative expression of *HvAP2L5* in developing spikes of the Wild-Type (AII) and *tw1* genotypes. Spikes were collected from 37-day-old plants and were approximately 1 cm long, each biological sample consisted of four pooled spikes. Expression was normalized using the geometric mean of two endogenous reference genes: ADP-ribosylation factor 1-like protein (*HvADP*; *HORVU.MOREX.r3.6HG0606370*) and non-coding small nucleolar RNA 14 (snoR14; *LOC123445868*). Asterisks indicate a significant difference from the Wild-Type, determined using a two-sided Dunnett's test: **$p < 0.01$.

Supplementary Figure 11. Sequence analysis of *tw.c1* CRISPR-Cas9 allele. Primer binding sites used for Sanger sequencing are highlighted in grey; guide RNA locations are highlighted in yellow; the resulting inversion lesion in the DNA is indicated in red highlighted font.

```
HvAP2L5    GTGACGAGGCAGCTGTTCCCCGCGCCGCCGCCCGCGCCCGGGGTCATGATG-GGGCAGGC   59
tw.c1      -------------------------------------GGGGGGGTCTGAATGGGGGCAGGC   24

HvAP2L5    GCCGGCGCCTCCGCCCACGGCGCCGGTGTGGCAACCTCGGCGCGCCGAGGAGCTGGTCGT  119
tw.c1      GCCGGCGCCTCCGCCCACGGCGCCGGTGTGGCAACCTCGGCGCGCCGAGGAGCTGGTCGT   84

HvAP2L5    GGCGCAGCGGGTGGCCCCCAAGAAGAAGACGCGGCGGGGGCCGAGGTCGCGCAGCTCGCA  179
tw.c1      GGCGCAGCGGGTGGCCCCCAAGAAGAAGACGCGGCGGGGGCCGAGGTCGCGCAGCTCGCA  144

HvAP2L5    GTACCGGGGCGTCACCTTCTACCGCAGGACCGGCCGCTGGGAGTCGCACATCTGGTTAGC  239
tw.c1      GTACCGGGGCGTCACCTTCTACCGCAGGACCGGCCGCTGGGAGTCGCACATCTGGTTAGC  204

HvAP2L5    CATCTCTTCTCGCCATGCCCCTCTACTTACCAAAAGAGTGAACCATTCATTAACTAATCA  299
tw.c1      CATCTCTTCTCGCCATGCCCCTCTACTTACCAAAAGAGTGAACCATTCATTAACTAATCA  264

HvAP2L5    ATCGCTGATTACACCAGGGATTGCGGGAAGCAGGTCTACTTGGGTGAGCTCAATCAAATC  359
tw.c1      ATCGCTGATTACACCAGGGATTGCGGGAAGCAGGTCTACTTGGGTGAGCTCAATCAAATC  324

HvAP2L5    CCAGCTCCAGCTCCCATCTTCCCCGTGTTTAATTTCGATTGTCTTTGCTATTATTATCAT  419
tw.c1      CCAGCTCCAGCTCCCATCTTCCCCGTGTTTAATTTCGATTGTCTTTGCTATTATTATCAT  384

HvAP2L5    CTTCTGCTAATTAAAGAAGTTGGTTGATTTCTTCAGGTGGTTTCGACACCGCGCACGCGG  479
tw.c1      CTTCTGCTAATTAAAGAAGTTGGTTGATTTCTTCAGGTGGTTTCGACACCGCGCACGCGG  444

HvAP2L5    CCGCAAGGTGAATAAATCATCATAAATTAATCAGGCTACGCTTGAACTCCTTTTACCCCG  539
tw.c1      CCGCAAGGTGAATAAATCATCATAAATTAATCAGGCTACGCTTGAACTCCTTTTACCCCG  504

HvAP2L5    ATTTGAGATTTGACCATGAACTGTGTACTGAGATGAGACGGTGTGGCGTGGCGCAGGGCC  599
tw.c1      ATTTGAGATTTGACCATGAACTGTGTACTGAGATGAGACGGTGTGGCGTGGCGCAGGGCC  564

HvAP2L5    TACGATCGCGCGGCGATCAAGTTCCGGGGGCTGGAGGCCGACATCAACTTCAATCTGAGC  659
tw.c1      TACGATCGCGCGGCGATCAAGTTCCGGGGGCGCACTT----GTGGAGCGTGACGCCGCGG  620

HvAP2L5    GACTACGAGG-------------------AGGATTTGAAGCAGGTAATCTTAT-GAAAGCC  700
tw.c1      TACTTCGAGCTCCCCCTGGCGAACCCGGTGCTCTGGCGGCGGAGGATATGCACGAACTCC  680

HvAP2L5    AAGCTAGATTGATTGCTGTGCCACCAAGTAGT-----GCGCCGGATGATGATAATATATG  755
tw.c1      TCCTTGGTCCAGTTCCTCATCTGGCAAAGAACACCACCCACCCACCGGATATCAACCATC  740

HvAP2L5    GGTGGTGTTGTCCGATGGTTGATATCCGGTGGGTGGGTGGTGTTTTTTGCCAGATGAGGA  815
tw.c1      GGACAACACCACCCATATATTATCATCATCCGG--CGCACTACTTGGTGGCACAGCAATC  798

HvAP2L5    ACTGGACCAAGGAGGAGTTCGTGCATATCCTCCGCCGCCAGAGCACCGGGTTCGCCAGGG  875
tw.c1      AATCTAGC----------------------TTGGCTTTCATAAGATTACCTGCTTCAAAT  836

HvAP2L5    GGAGCTCGAAGTACCGCGGCGTCACGCTCCACAAGTGCGGCCGCTGGGAGGCAAGGATGG  935
tw.c1      CCTCCTCGTAGTCGCTCAGATTGAAGTTGATGTCGGCCTCCAGCTGGGAGGCAAGGATGG  896

HvAP2L5    GCCAACTGCTCGGCAAGAAGTAAGCAGACTACACACACGCTAAAATTATATTAACTTCAT  995
tw.c1      GCCAACTGCTCGGCAAGAAGTAAGCAAACTACACACACGCTAAAATTATATTAACTTCAT  956

HvAP2L5    TCACATTATCATAATTTCCTTAATCAATGCCATATACTGCAAGATTAGG            1042
tw.c1      TCACATTATCATAATTTCCTTAATCAATGCCATATACTGCAAAAATAAG            1016
```

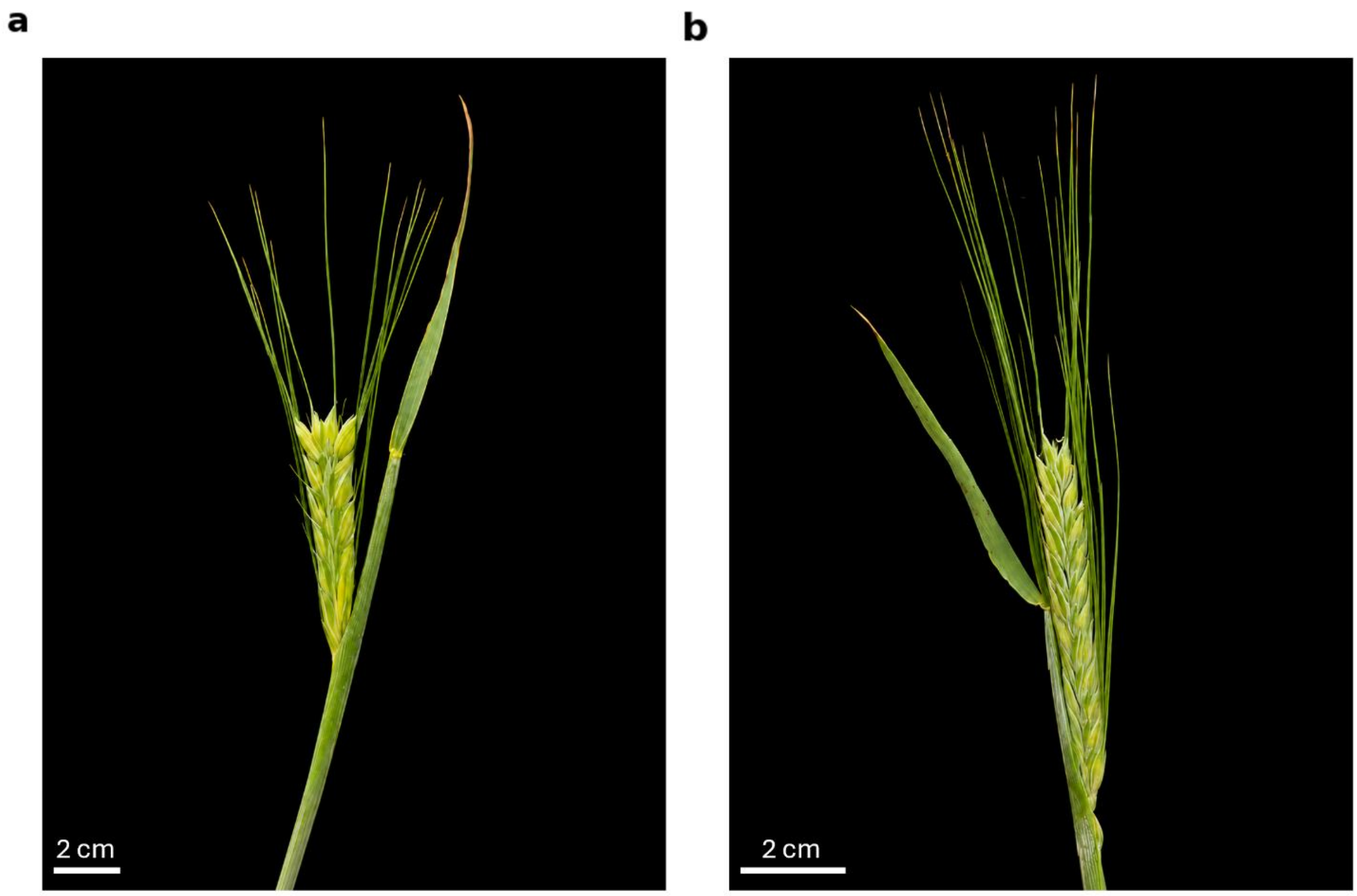

Supplementary Figure 12. Representative photos of 75-day old spike from wild-type (A) and tw.c1 (B) plants. Scale bar = 2 cm.

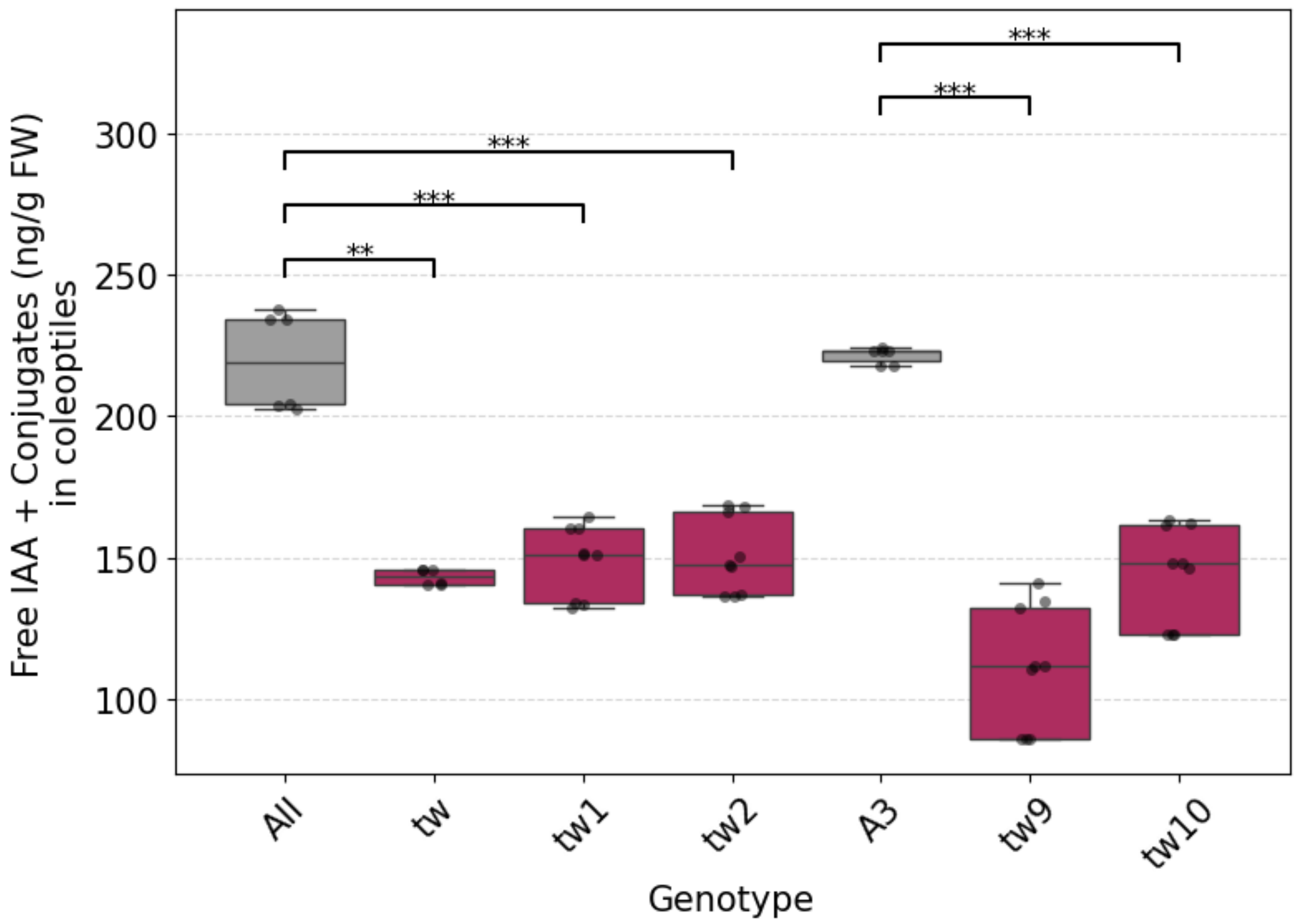

Supplementary Figure 13. Free IAA and IAA conjugate levels in coleoptiles of *tw* mutants and their corresponding Wild-Type genotypes, determined using high-performance thin-layer chromatography (HPTLC). Sample sizes: AII n = 6; *tw* n = 6; *tw1* n = 9; *tw2* n = 10; A3 n = 6; *tw9* n = 9; *tw10* n = 9. Differences were assessed using Mann–Whitney U test, followed by Benjamini–Hochberg correction for multiple comparisons. Asterisks indicate significant differences compared to the corresponding WT: **p < 0.01, ***p < 0.001.

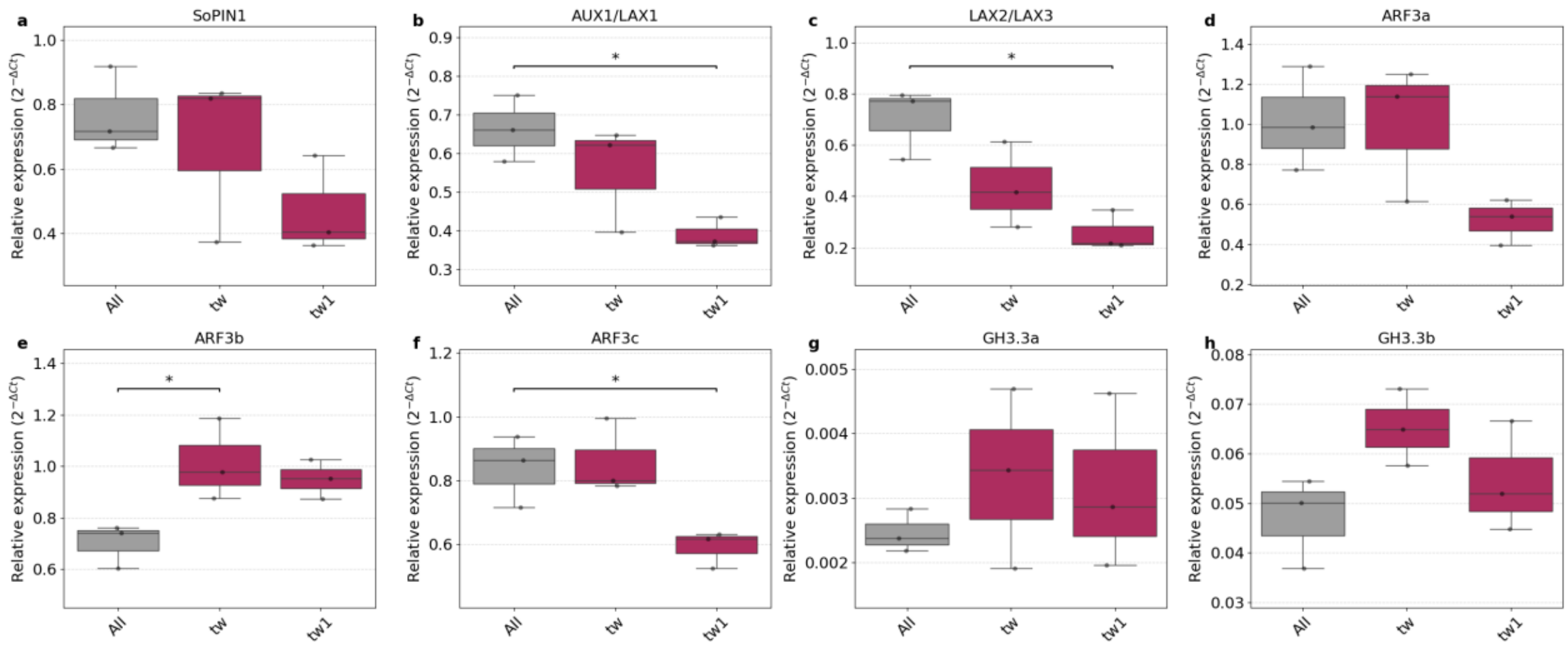

Supplementary Figure 14. Expression of genes involved in auxin transport, signalling and homeostasis in developing spikes of AII, *tw* and *tw1* genotypes. Spikes were collected from 37-day-old plants and were approximately 1 cm long, each biological sample consisted of four pooled spikes. Transcript levels of (a) *SoPIN1 (HORVU.MOREX.r3.4HG0356750)*, (b) *AUX1/LAX1 (HORVU.MOREX.r3.3HG0301040)*, (c) *LAX2/LAX3 (HORVU.MOREX.r3.4HG0392470)*, (d) *ARF3a (HORVU.MOREX.r3.1HG0077510)*, (e) *ARF3b (HORVU.MOREX.r3.1HG0087670)*, (f) *ARF3c (HORVU.MOREX.r3.3HG0289370)*, (g) *GH3.3a (HORVU.MOREX.r3.3HG0291280)* and (h) *GH3.3b (HORVU.MOREX.r3.3HG0294170)* were analysed by RT-qPCR. Expression was normalized using the geometric mean of two endogenous reference genes: ADP-ribosylation factor 1-like protein (*HvADP*; *HORVU.MOREX.r3.6HG0606370*) and non-coding small nucleolar RNA 14 (*snoR14*; *LOC123445868*). Asterisks indicate a significant difference from the Wild-Type, determined using a two-sided Dunnett's test: *$p < 0.01$.

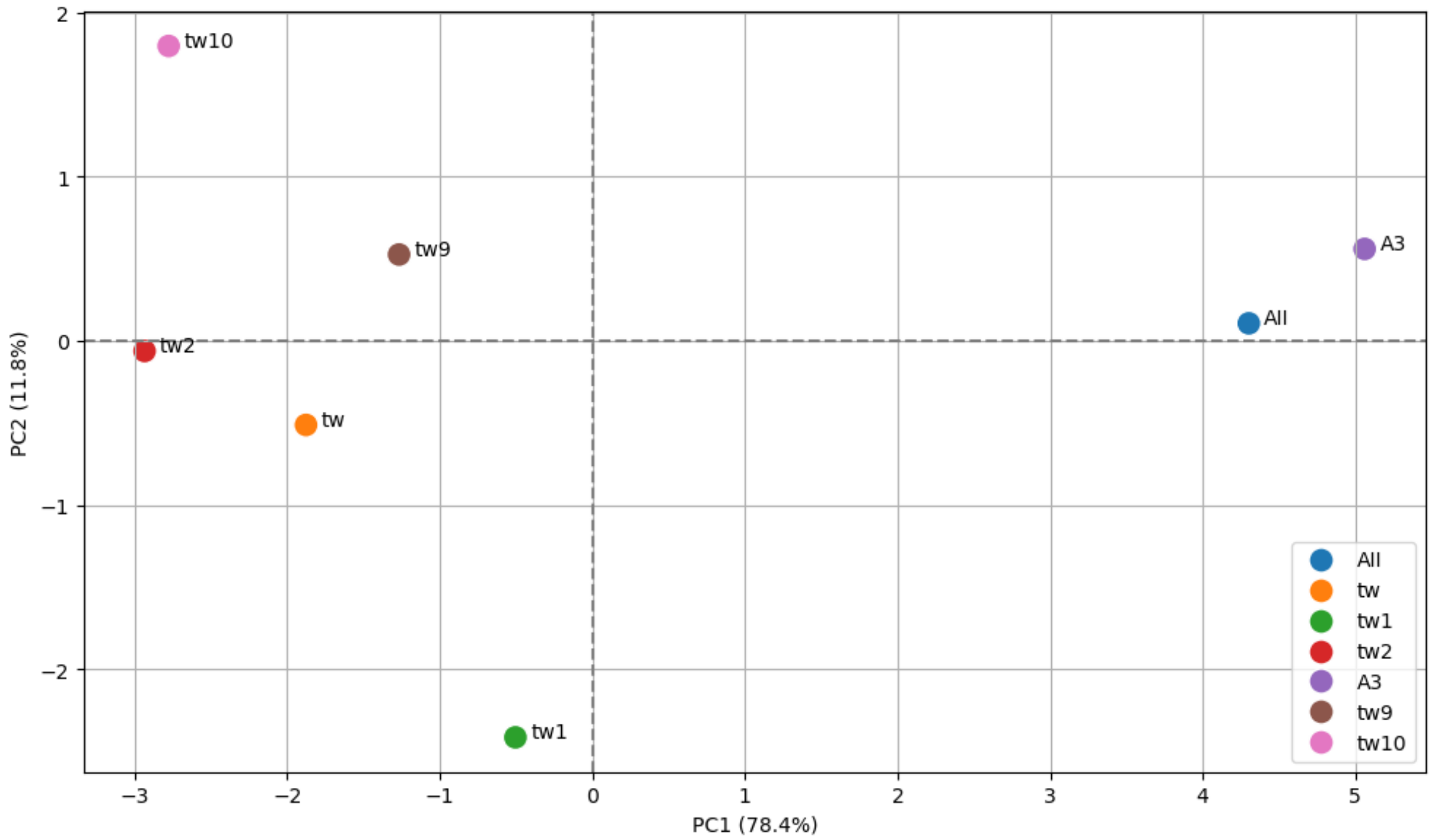

Supplementary Figure 15. PCA created from agronomic (spike length (cm), awn length, grain number per spike, tiller count per plant, plant height (cm) and sterile spikelet count per spike) and spike/flower morphometric (frequency of lodicule to reproductive organ transformation, frequency of deviation in the number of floral organs, frequency of sterile spike base, frequency of spikeletless rachis node/ elongated internode (spike gap), frequency of presence of two spikelets at the top of the spike, and frequency of presence of a crowned spike tip) parameters. WTs: AII and A3. tw-type mutants: *tw*, *tw1*, *tw2*, *tw9* and *tw10.*

Supplementary Table 1. Oligonucleotides used for amplification of *HvAP2L5* gene fragments and single-guide RNAs used for CRISPR/Cas9-mediated editing of *HvAP2L5*.

Purpose	Gene/name	Direction	Primers/oligonucleotides (5′->3′) used in this research
sgRNA	Position 3.1 (T1)	Forward	CTTGCTTGTGGTTGGGCACCTTTGAGA
		Reverse	AAACGTGTGGCAACCTCGGCGCG
	Position 4.1 (T2)	Forward	CAGACTTGTCGCGCAGCTCGCAGTACCGTTT
		Reverse	TCTAAAACGGTACTGCGAGCTGCGCGACAAG
	Position 3.2 (T3)	Forward	CTTGATCAAGTTCCGGGGGCTGG
		Reverse	AAACCCAGCCCCCGGAACTTGAT
	Position 4.2 (T4)	Forward	CAGACTTGCTCCACAAGTGCGGCCGCTGTTT
		Reverse	TCTAAAACAGCGGCCGCACTTGTGGAGCAAG
Targeted mutation validation	*AP2L5*	Forward	GTGACGAGGCAGCTGTT
		Reverse	CCTAATCTTGCAGTATATGGCATTG
T-DNA insertion validation	*hpt*	Forward	GGATTTCGGCTCCAACAATG
		Reverse	TATTGGGAATCCCCGAACATC
Candidate gene validation	*AP2L5*/W1	Forward	GACACGTGGCGTCCGCA
		Reverse	GTGGGTGGCCGTAGCGATTA
	AP2L5/W2	Forward	GTCACCTTCTACCGCAGGAC
		Reverse	TGCCCTGTCACATCTATACCC
	AP2L5/W3	Forward	GATGAGGAACTGGACCAAGGAG
		Reverse	TCCAAGTCAAGAGCATCAGCAT
	AP2L5/W4	Forward	CGCTCACTCACTCTCTTGTCAT
		Reverse	CTACTAGCTCACAGGGCAAACA
	AP2L5/W5	Forward	CATTGGCGTCTCTCGATCTCA
		Reverse	AACGCAACAATGGTGATGGTG
Upstream region quantificat ion	US_1	Forward	ATGTTTGGTTAGATCGATGACACGG
		Reverse	CCCAAGCGCATCATCAGAGG
	US_2	Forward	CTTGGTGTCTGTGTAGGTGTAG
		Reverse	GATCTCCGATGGTATGGCATTAT
	US_3	Forward	GTCAAATCAAGCAACGCAACGG
		Reverse	AAATTAATGGCGGCAGGTTCA

Supplementary Table 2. Primers used for qPCR analyses.

Gene ID	Gene/name	Direction	Primers/oligonucleotides (5′->3′) used in this research
HORVU.MOREX.r3.6HG0606370	*Endogenous control, HvADP*	Forward	GCTCTCCAACAACATTGCCAAC
		Reverse	GAGACATCCAGCATCATTCATTCC
LOC123445868	*Endogenous control,* snoR14	Forward	GATGTTTATGTATGATAGTCTGTC
		Reverse	GTCGGGATGTATGCGTGTC
HORVU.MOREX.r3.5HG0525620	*HvAP2L5*	Forward	TCGACAGCGAAGTTGAAGC
		Reverse	TCAAAGTTAGTCACAGCATCCC
HORVU.MOREX.r3.4HG0356750	*soPIN1*	Forward	CACCCTCGTCTTCTACTTGTTC
		Reverse	CTGGCTGGGTGAAGAAAGAA
HORVU.MOREX.r3.3HG0301040	*AUX1/LAX1*	Forward	CTACCTCATCAGCGTCCTCTA
		Reverse	CACTGGATGACGTGGTTCTT
HORVU.MOREX.r3.4HG0392470	*LAX2/LAX3*	Forward	CTGTTGCTTCCCTTCTCCAT
		Reverse	CCGAAGGTGTAGAGGATGTTG
HORVU.MOREX.r3.1HG0077510	*ARF3a*	Forward	GGTGAACTGAGATTGGGTGTAA
		Reverse	ACTGGCAACAGCAGATAGTG
HORVU.MOREX.r3.1HG0087670	*AF3b*	Forward	CTCATCTTGGTTCGGGAGAAG
		Reverse	GCCAGGGAGATGCCAAATA
HORVU.MOREX.r3.3HG0289370	*ARF3c*	Forward	GTCAACCACGTAGGCATCTT
		Reverse	CCATCATCACCACGGAGAAATA
HORVU.MOREX.r3.3HG0291280	*GH3.3a*	Forward	GGGCTCTACTTCCTCTTCATC
		Reverse	GAAGTGGTCGCTCTTGTAGTA
HORVU.MOREX.r3.3HG0294170	*GH3.3b*	Forward	GTTCCCACCATGTGCTACTT
		Reverse	GCTTCACATCCACTAGATCGAC

Supplementary Table 3. Morphometric traits of *tw* barley mutants and Wild-Types: mean, 95% confidence intervals (CI), n.

Genotype	Spike length (cm)	Awn length (cm)	Grain count per spike	Empty spikelets	Plant height (cm)	Tiller count
**AII**	7.51 [7.25, 7.77] (n=30)	15.31 [14.50, 16.13] (n=30)	20.80 [19.84, 21.76] (n=30)	2.33 [1.77, 2.89] (n=30)	91.50 [89.79, 93.21] (n=30)	9.07 [8.03, 10.10] (n=30)
**tw**	5.45 [5.18, 5.73] (n=30)	12.89 [12.23, 13.54] (n=30)	12.53 [11.13, 13.93] (n=30)	3.73 [3.27, 4.19] (n=30)	94.40 [92.96, 95.84] (n=30)	11.50 [10.37, 12.63] (n=30)
**tw1**	5.53 [5.22, 5.84] (n=30)	12.38 [11.49, 13.26] (n=30)	13.53 [12.23, 14.84] (n=30)	3.47 [2.88, 4.05] (n=30)	90.30 [88.60, 92.00] (n=30)	10.97 [9.63, 12.31] (n=30)
**tw2**	4.77 [4.51, 5.03] (n=30)	10.61 [9.80, 11.41] (n=30)	11.67 [10.65, 12.68] (n=30)	3.40 [2.67, 4.13] (n=30)	94.50 [93.23, 95.77] (n=30)	12.03 [10.92, 13.14] (n=30)
**A3**	7.67 [7.26, 8.09] (n=30)	16.22 [15.52, 16.91] (n=30)	23.13 [22.00, 24.27] (n=30)	1.27 [0.86, 1.68] (n=30)	92.39 [90.02, 94.77] (n=28)	8.11 [6.97, 9.24] (n=28)
**tw9**	5.31 [5.03, 5.60] (n=30)	11.94 [11.01, 12.86] (n=30)	12.80 [11.73, 13.87] (n=30)	3.17 [2.71, 3.63] (n=30)	95.57 [93.69, 97.44] (n=30)	10.63 [9.33, 11.94] (n=30)
**tw10**	5.05 [4.78, 5.33] (n=30)	11.30 [10.31, 12.30] (n=30)	11.70 [10.72, 12.68] (n=30)	3.67 [3.18, 4.16] (n=30)	95.83 [94.34, 97.32] (n=30)	11.57 [10.56, 12.57] (n=30)

Supplementary Table 4. Comparison between WT and mutant's morphometric parameters. First value: p value of Mann–Whitney U test, second value: p value of Mann–Whitney U test, followed by Benjamini–Hochberg correction for multiple comparisons.

Genotype	Spike length (cm) p-values	Awn length (cm) p-values	Grain count per spike p-values	Empty spikelets p-values	Plant height (cm) p-values	Tiller count p-values
**AII vs tw**	9.14491E-11; 2.28623E-10	1.59781E-05; 1.59781E-05	6.65701E-10; 8.32126E-10	0.000252321; 0.000420535	0.012779909; 0.021299849	0.002253104; 0.003755173
**AII vs tw1**	8.94585E-10; 8.94585E-10	4.95634E-06; 6.19542E-06	1.37623E-09; 1.37623E-09	0.005150743; 0.006438429	0.542749104; 0.542749104	0.037675355; 0.037675355
**AII vs tw2**	3.69588E-11; 1.84794E-10	1.23268E-08; 6.16338E-08	4.85019E-11; 1.13634E-10	0.019632297; 0.019632297	0.006509858; 0.016274644	8.92784E-05; 0.00039864
**A3 vs tw9**	7.2832E-10; 8.94585E-10	2.16934E-07; 3.61556E-07	6.81804E-11; 1.13634E-10	4.65181E-07; 1.16295E-06	0.031964004; 0.039955005	0.008258961; 0.010323701
**A3 vs tw10**	2.32966E-10; 3.88277E-10	3.42544E-08; 8.5636E-08	4.36111E-11; 1.13634E-10	4.64301E-08; 2.3215E-07	0.006481757; 0.016274644	0.000159456; 0.00039864

Supplementary Table 5. Inflorescence phenotypes of *tweaky spike* (*tw*) barley mutants and their respective Wild-Type backgrounds.

Genotype	Spike count (n)	Frequency of sterile spike base	Frequency of spike gaps	Frequency of presence of two spikelets at the top of the spike	Frequency of presence of a crowned spike tip
AII	62	0	0	0.048387	0
tw	70	0.742857	0.2	0.571429	0.3
tw1	62	0.870968	0	0.596774	0.290323
tw2	70	0.885714	0.2	0.514286	0.285714
A3	61	0.098361	0.016393	0.081967	0
tw9	63	0.571429	0.111111	0.68254	0.174603
tw10	61	0.606557	0.131148	0.721311	0.114754

Supplementary Table 6. Flower phenotypes of *tweaky spike* (*tw*) barley mutants and their respective Wild-Type backgrounds.

Genotype	Spikelet count (n)	Frequency of lodicule transformation	Frequency of deviation in the number of flower organs
AII	62	0	0.016129
tw	73	0.60274	0.041096
tw1	81	0.320988	0.061728
tw2	98	0.72449	0.132653
A3	94	0	0
tw9	73	0.589041	0.068493
tw10	77	0.87013	0.25974

Supplementary Table 7. Comparison between WT and mutant's inflorescence/floret phenotype frequencies. Differences in phenotype frequencies were assessed using a two-proportion Z-test.

Comparison	Lodicule transformation p-value	Deviation in the number of flower organs p-value	Sterile spike bottom p-value	Spike gaps p-value	Paired spikelets on the top of the spike p-value	Crowned spikes p-value
AII vs tw	9.64E-14	0.393906	0	0.000196	1.56E-10	2.56E-06
AII vs tw1	8.14E-07	0.177719	0		6.51E-11	4.46E-06
AII vs tw2	0	0.011046	0	0.000196	4.76E-09	4.9E-06
AII vs A3	NaN	0.216727	0.011341	0.311401	0.450192	NaN
AII vs tw9	2.47E-13	0.141223	1.74E-12	0.006905	1.97E-13	0.00057
AII vs tw10	0	6.7E-05	2.24E-13	0.003188	1.6E-14	0.006021
tw vs tw1	0.000454	0.56455	0.064741	0.000196	0.768157	0.903197
tw vs tw2	0.093342	0.042004	0.029756	1	0.497378	0.852666
tw vs A3	0	0.047328	1.28E-13	0.000995	3.99E-09	3.04E-06
tw vs tw9	0.866071	0.467034	0.03695	0.160409	0.186526	0.09117
tw vs tw10	0.00019	0.000203	0.095446	0.29299	0.074411	0.009883
tw1 vs tw2	6.93E-08	0.116305	0.795574	0.000196	0.34143	0.953462
tw1 vs A3	2.63E-09	0.014525	0	0.311401	1.75E-09	5.25E-06
tw1 vs tw9	0.000838	0.864922	0.000192	0.006905	0.317887	0.125406
tw1 vs tw10	2.36E-12	0.000652	0.000832	0.003188	0.145315	0.015553
tw2 vs A3	0	0.000255	0	0.000995	1.02E-07	5.75E-06
tw2 vs tw9	0.063101	0.176298	3.96E-05	0.160409	0.04851	0.130206
tw2 vs tw10	0.019172	0.03289	0.000208	0.29299	0.015348	0.015825
A3 vs tw9	0	0.009988	2.63E-08	0.031844	6.69E-12	0.000629
A3 vs tw10	0	1.45E-07	4.23E-09	0.015331	5.91E-13	0.006428
tw9 vs tw10	9.98E-05	0.001681	0.691049	0.732303	0.637113	0.344234

Supplementary Table 8. Spike phenotypes of *tw.c1* barley T0 mutant and T0 plant regenerated after transformation, carrying the same T-DNA as *tw.c1* line, but has no change in *HvAP2L5* gene. Differences in phenotype frequencies were assessed using Mann–Whitney U test.

Genotype	Spike count (n)	Mean spike length (cm)	Mean grain count per spike	Frequency of two spikelets at the top of the spike	Frequency of crowned spike tip
tw.c1	90	5.05	6.15	0.24	0.22
non-edited sibling line	39	4.80	7.84	0	0.026
P value		0.342902	0.000774	0.000698	0.005476

Supplementary Table 9. Flower phenotypes of *tw.c1* barley mutant and their WT 'Golden promise' (GP). Differences in phenotype frequencies were assessed using a two-proportion Z-test.

Genotype	Spikelet count (n)	Frequency of lodicule transformation	Frequency of deviation in the number of flower organs
GP	53	0	0
tw.c1	42	0.12	0.047
P value		0.009553	0.110724
